# Single-nucleus epigenomic profiling of the adult human central nervous system unveils epigenetic memory of developmental programs

**DOI:** 10.1038/s41593-026-02208-0

**Published:** 2026-03-19

**Authors:** Mukund Kabbe, Eneritz Agirre, Karl E. Carlström, Özge Dumral, Yuk Kit Lor, Fabio Baldivia Pohl, Nicolas Ruffin, David van Bruggen, Mandy Meijer, Luise A. Seeker, Nadine Bestard-Cuche, Alex R. Lederer, Jilin Zhang, Virpi Ahola, Steven A. Goldman, Erik Edström, Lisa Arvidsson, Tiago Holm Moreira, Marek Bartosovic, Maja Jagodic, Anna Williams, Gonçalo Castelo-Branco

**Affiliations:** 1 Laboratory of Molecular Neurobiology, Department of Medical Biochemistry and Biophysics, Science for Life Laboratory, Karolinska Institutet, Stockholm, Sweden; 2 Department of Clinical Neuroscience, Centre for Molecular Medicine, Karolinska Institutet, Karolinska University Hospital, Stockholm, Sweden; 3 Max Delbrück Centre for Molecular Medicine in the Helmholtz Association (MDC Berlin), Berlin, Germany; 4 Centre for Regenerative Medicine, Institute of Regeneration and Repair, University of Edinburgh, Edinburgh, UK; 5 Department of Bioengineering, Stanford University, Stanford, CA USA; 6 Laboratory of Brain Development and Biological Data Science, School of Life Sciences, Ecole Polytechnique Federale de Lausanne, Lausanne, Switzerland; 7 Ming Wai Lau Centre for Reparative Medicine, Karolinska Institutet, Hong Kong, China; 8 Institute of Biomedicine, University of Eastern Finland, Kuopio, Finland; 9 Centre for Translational Neuromedicine, University of Copenhagen, Copenhagen, Denmark; 10 Centre for Translational Neuromedicine, University of Rochester, Rochester, NY USA; 11 Department of Clinical Neuroscience, Karolinska Institutet, Solna, Sweden; 12 Department of Women’s and Children’s Health, Karolinska Institutet, Solna, Sweden; 13 Department of Neurology, Karolinska University Hospital, Stockholm, Sweden; 14 Department of Biochemistry and Biophysics, Stockholm University, Stockholm, Sweden; 15 Present Address: NEXUS Epigenomics AB, Stockholm, Sweden

**Keywords:** CNS cancer, Chromatin analysis, Epigenetics in the nervous system

## Abstract

Neural cells in the adult human central nervous system (CNS) display extensive transcriptional heterogeneity. How different layers of epigenetic regulation underpin this heterogeneity is poorly understood. Here we profile, at the single-nuclei epigenomic level, distinct regions of the adult human CNS, for chromatin accessibility and simultaneously for the histone modifications H3K27me3 and H3K27ac. We unveil a putative *SOX10* enhancer and primed chromatin signatures at HOX loci in spinal-cord-derived human oligodendroglia (OLG) and astrocytes, but not microglia. These signatures in adult OLG were reminiscent of developmental profiles but were decoupled from robust gene expression. Moreover, using high-resolution Micro-C, we show that induced pluripotent stem-cell-derived human OLGs exhibit a HOX chromatin architecture compatible with the primed chromatin in adult OLGs, bearing a strong resemblance not only to OLG developmental architecture but also to high-grade pontine gliomas. Thus, epigenetic memory from developmental states in adult OLG not only enables them to promptly transcribe Hox family genes during regeneration but also makes them susceptible to gliomagenesis.

## Abstract (web summary)

Single-nucleus epigenomic maps of the adult human brain and spinal cord reveal that adult oligodendroglia retain developmental chromatin patterns, suggesting a molecular memory that may shape repair processes and cancer vulnerability.

## Main

The human central nervous system (CNS) contains diverse neuronal and glial populations that together support sensory processing, motor control and higher cognitive function. Mature oligodendrocytes (MOLs) are a glial cell type that wraps neuronal axons with myelin, enabling rapid saltatory conduction^1–3^. MOLs are primarily found within the white matter (WM) areas of the CNS, whereas their progenitor population, oligodendrocyte precursor cells (OPCs), are uniformly distributed throughout the CNS. Previous studies that investigate the oligodendroglial (OLG) lineage have identified region-specific transcriptomic differences in the human CNS^4,5^.

While the transcriptome of human neural populations has been well characterized, the underlying regulatory chromatin landscape remains largely unknown. Chromatin accessibility provides a snapshot of the regulatory blueprint underlying cell states^6,7^ and has been used to identify organ-specific regulatory elements^8,9^, developmental regulatory circuits^9,10^ and disease-associated effects^11–16^. However, chromatin accessibility represents one regulatory layer. Histone post-translational modifications (PTMs) and DNA modifications also have essential roles in transcriptional regulation^17^. Single-cell studies of DNA methylation^18^ and individual histone marks^19–29^ have started elucidating epigenetic states in the adult mouse CNS, but similar characterization of the adult human CNS remains limited. In humans, DNA methylation and chromatin architecture have been analyzed at single-cell resolution^30^. Single-cell H3K27me3 profiling has been performed in a glioblastoma sample^23^ alongside bulk analyses^11^, and several histone modifications in sorted CNS subpopulations^31,32^. Recent multimodal assays have enabled joint single-cell capture of H3K27ac/RNA in the mouse brain and human M1 cortex^25,33^. However, joint single-cell profiling of multiple histone modifications across the adult human CNS is still lacking.

Here we provide a single-cell chromatin accessibility dataset of the adult human CNS across three anterior–posterior axis regions, together with a joint multimodal single-cell histone PTM dataset in the adult human CNS. These resources, available at the UCSC Cell Browser and UCSC Genome Browser^34^ (https://cns-nanocuttag-atac.cells.ucsc.edu), define H3K27me3 and H3K27ac landscapes across major cell populations across regions, and enabled the identification of a putative *SOX10* enhancer and lineage-specific transcription factor (TF) regulatory networks. We show that adult OLGs show chromatin accessibility, histone PTM patterns and three-dimensional (3D) architecture reminiscent of their developmental counterparts. This suggests that epigenetic memory of key developmental genes persists in adult OLGs, potentially priming them for rapid activation of HOX loci during regenerative responses, while also making them susceptible to hijacking in tumor transformation, such as gliomagenesis.

## Results

### Single-nucleus assay for transposase-accessible chromatin using sequencing of the adult human CNS reveals differential chromatin and TF motif accessibility in different CNS cell types

**Fig. 1:**
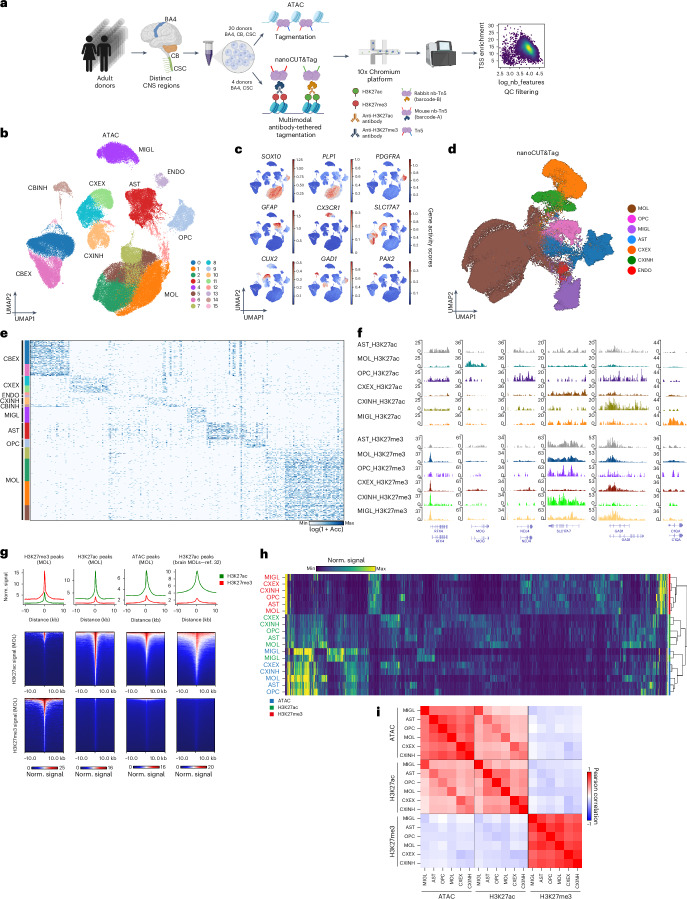
snATAC–seq and H3K27ac/H3K27me3 nanoCUT&Tag identify major human cell types in the CNS. **a**, Schematic for snATAC–seq and nanoCUT&Tag experiments in adult human CNS tissue. **b**, Two-dimensional UMAP of the snATAC dataset colored by clusters and labeled by cell type. **c**, Gene activity scores for different genes in the identified cell types. **d**, Two-dimensional UMAP of the H3K27ac nanoCUT&Tag dataset with cell annotations based on integration with snATAC–seq. **e**, Heatmap showing snATAC–seq differentially accessible peaks across different clusters and cell types. **f**, nanoCUT&Tag genome browser snapshot showing H3K27ac (top) and H3K27me3 (bottom) pseudobulk signal distribution across different marker genes for each cell type. **g**, nanoCUT&Tag meta-signal enrichment plots for H3K27ac (green) and H3K27me3 (red) in the MOL population. Top: line plots showing signal enrichment for the two modalities at different peak sets. Middle and bottom: heatmap showing H3K27ac (middle) and H3K27me3 (bottom) signal enrichment across different peak sets. Peak sets (left to right)—H3K27me3 peaks, H3K27ac peaks, ATAC peaks and ATAC peaks from a previously published dataset^32^. **h**, Trimodal clustering of the genome highlights patterns of signal distribution across all cell types. **i**, Correlation matrix of ATAC, H3K27ac and H3K27me3 signals in each cell type shows strong correlation between active marks for individual cell types, and anticorrelation with the repressive H3K27me3. UMAP, uniform manifold approximation and projection; norm., normalized. Schematic in **a** created in BioRender; Castelo-Branco, G. https://biorender.com/orfbpyi (2025).

**Extended Data Fig. 1:**
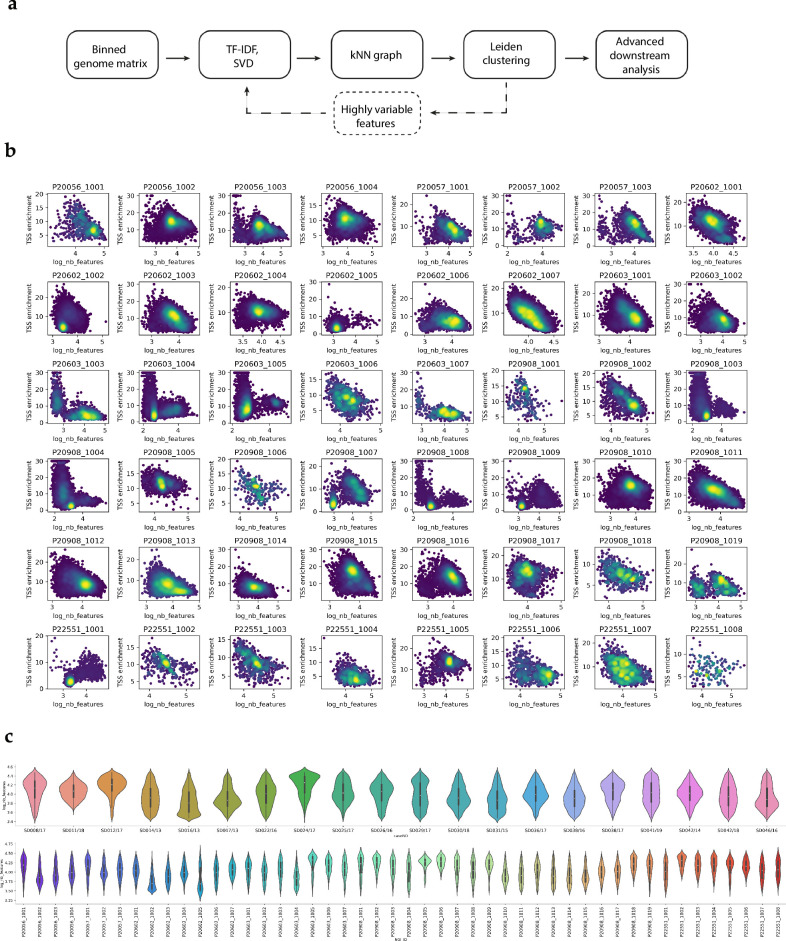
QC and preprocessing of the snATAC-seq dataset. **a**, Schematic showing downstream data analysis workflow. **b**, Density scatter QC plots for all 48 samples in the snATAC-seq dataset. Number of unique fragments (log scale) on x axis and TSS enrichment score on y axis. **c**, Violin plots showing number of unique fragments (log) from each donor (top) and each sample (bottom). Data in each violin plot composed of all cells within that donor (top) or sample (bottom). Black box within each violin plot represents the interquartile range (IQR), white dot is the median, and whiskers extend to 1.5× IQR.

**Extended Data Fig. 2:**
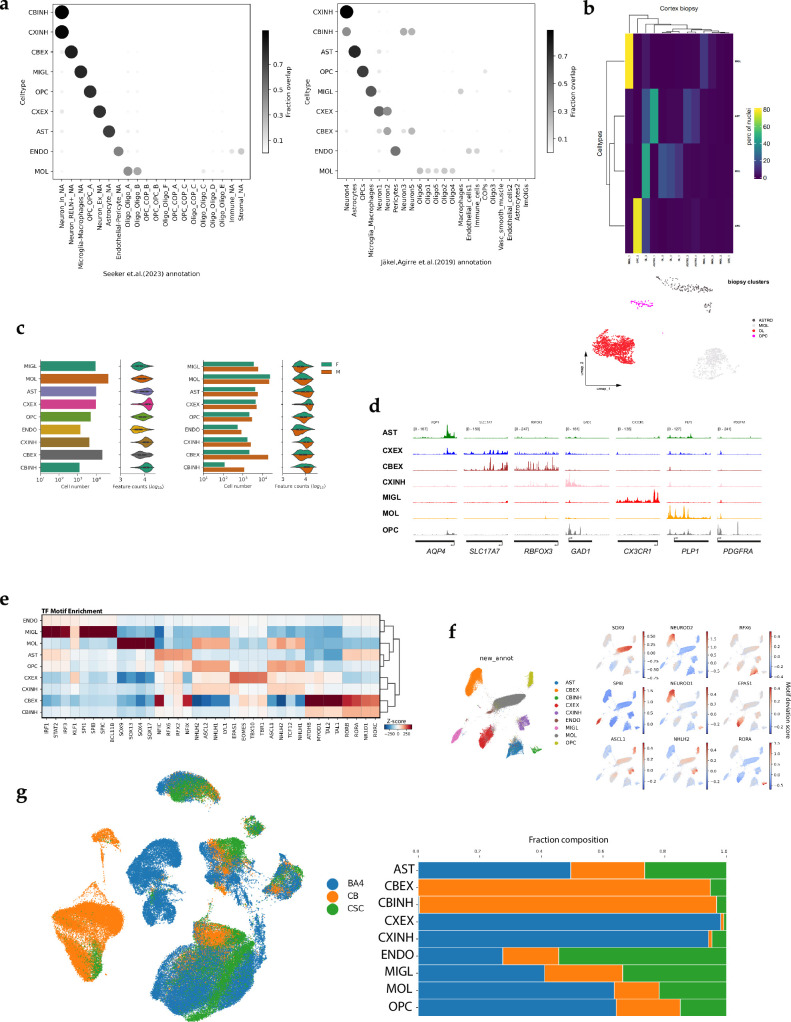
Validation of cell-type annotations through integration with external datasets. **a**, Correlation plot showing quality of cell type annotations using the metagene scores (y axis) and annotations from integration with previously published datasets (ref. ^37^—right, ref. ^5^—left). **b**, Correlation plot showing quality of cell type annotations from the snATAC-seq dataset when integrated with a human cortical biopsy dataset (this study), and the cortical biopsy clusters in a 2D-UMAP embedding (bottom). **c**, Bar chart and violin plots showing cell numbers and feature counts (log scale) for each cell type (left) and contribution of each sex to the metrics (right). Data from each plot are composed of total cell counts for individual cell types (left) and the sex distribution for those cells (right). See Supplementary Table 1 for detailed information on donor metadata. Black box within each violin plot represents the interquartile range (IQR), white dot is the median, and whiskers extend to 1.5× IQR. **d**, snATAC-seq genome coverage tracks for different marker genes in each identified cell type. All columns are group normalized (range is shown in the first panel for each column). Gene location and TSS orientation are shown below. **e**, TF motif enrichment matrix showing the top four TF motifs identified as being differentially accessible in each cell type. **f**, 2D UMAP showing cell clustering based on the chromVAR calculated TF motif deviations. Marker TF enrichment for each cell type is shown on the right. **g**, 2D UMAP (left) and bar chart (right) showing tissue composition of cell types in the snATAC-seq dataset.

We collected a cohort of 60 tissue samples from 20 postmortem donors ranging in age from 34 to 74 years old, with equal representation of both sexes, and frozen tissue samples from three distinct regions of the CNS from each donor—primary motor cortex (Brodmann area 4 (BA4)), cerebellum (CB) and cervical spinal cord (CSC; Supplementary Table 1). Based on tissue quality metrics^5^ (Methods), we isolated WM-dominant areas from the tissue, dissociated them into single-nuclei suspensions and performed single-nucleus assay for transposase-accessible chromatin using sequencing (snATAC–seq) using the 10x Genomics Chromium platform (Fig. 1a). After sequencing and stringent quality control (QC) based on the number of unique reads and per-cell transcription start site (TSS) enrichment score (Extended Data Fig. 1a–c), we retained 108,626 nuclei representing all three regions (motor cortex = 55,037 cells, CB = 34,819 cells, CSC = 18,770 cells), with a median of 8,154 fragments per cell, and a median TSS enrichment (TSSe) score of 10.7 (Extended Data Fig. 1b). We built a binned genome count matrix^35,36^ followed by latent semantic indexing to obtain 16 distinct clusters across the three regions (Fig. 1b,c). To annotate cell types, we constructed a gene-activity matrix and assigned a metagene score to the broad cell types present in the CNS^22^ (Methods). Using the scores, we identified all major cell types, including cerebellar excitatory neurons (CBEX = 21,801 cells), cerebellar inhibitory neurons (CBINH = 1,301 cells), cortical excitatory neurons (CXEX = 9,671 cells) and cortical inhibitory neurons (CXINH = 4,353 cells). Conversely, the glial cell populations were relatively homogeneous, and we identified MOLs (MOL = 45,325 cells), OPCs (OPC = 5,130 cells), microglia (MIGL = 9,528 cells) and astrocytes (AST = 10,098 cells). We also found pericytes/endothelial cells (ENDO = 1,419 cells; Extended Data Fig. 2c,d). We identified marker genes for the different populations including *SOX10* for the OLG lineage, *PDGFRA* for OPCs, *PLP1* for MOLs, *AQP4* for AST, *AIF1* for MIGL, *RBFOX3* for excitatory neurons and *GAD1* for inhibitory neurons (Fig. 1c and Extended Data Fig. 2d). We confirmed the validity of our metagene-derived annotations by integrating the gene activity object with a paired single-nuclei transcriptomic dataset of the same cohort^5^, a previously published dataset^37^ and a prefrontal adult prefrontal cortex biopsy dataset that we generated for benchmarking (Extended Data Fig. 2a,b).

We then investigated TF motif accessibility differences in the different cell types in our dataset, using chromVAR^38^. Clustering on the motif deviations identified marker TFs for the different populations, for instance SOX TFs for OLG, or a specific RORA enrichment in the cerebellar neurons, which is required for cerebellar Purkinje cell maturation^39^ (Extended Data Fig. 2e,f). We observed lower correlation in endothelial cell annotations between our dataset and the integrated reference datasets (Extended Data Fig. 2a,b). This reduced correlation likely stems from a combination of biological and technical factors, including the inherent low representation of endothelial cells in the dataset and reduced chromatin coverage for this cell type in snATAC–seq data. Consequently, the downstream motif enrichment analysis (Extended Data Fig. 2e) did not reveal clear motif enrichments for endothelial cells, while it is clear for other cell types. Interestingly, while the neurons clustered distinctly according to region and broad electrophysiological profiles, we did not observe the same distinctions within the glial populations (Extended Data Fig. 2g), suggesting that the chromatin states of glial cells may be more region-agnostic and present plasticity to account for their varied functions.

**Extended Data Fig. 3:**
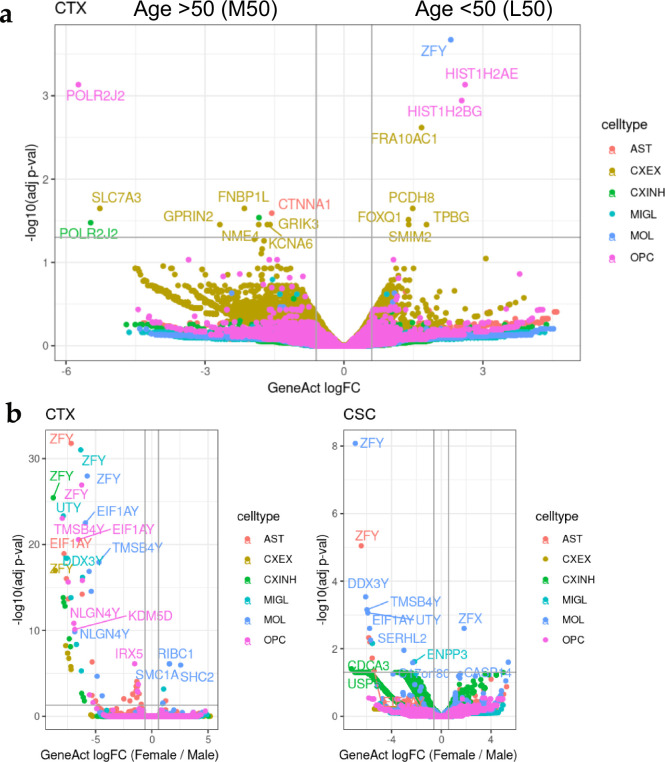
Differential accessibility across age and sex in major CNS cell types. **a**, Volcano plot showing differentially accessible genes in snATAC-seq based on age (less than or greater than 50 years old) in the different cell types. Two-sided t-test with Benjamini–Hochberg correction. Thresholds: adjusted P-value: 0.001, logFC = 1.5. **b**, Volcano plots showing differentially accessible genes in snATAC-seq based on sex in different cell types in the cortex (left) and spinal cord (right). Two-sided t-test with Benjamini–Hochberg correction. Thresholds: adjusted P-value: 0.001, logFC = 1.5.

To investigate whether chromatin accessibility changes with age, we analyzed differential accessibility across major cell types in donors younger than 50 (L50) and older than 50 (M50) years. Although age-associated changes were most evident in the neuronal populations, especially in CXEX population (Extended Data Fig. 3a and Supplementary Table 4), the number of nuclei per cell type, region and age group was too small to provide statistical power for additional comparisons and strong conclusions considering the age variable. Sex-based differences were primarily restricted to gonosomal genes (Extended Data Fig. 3b and Supplementary Table 4).

### Single-nucleus nanoCUT&Tag H3K27ac and H3K27me3 profiling of major cell populations in the adult human CNS

**Extended Data Fig. 4:**
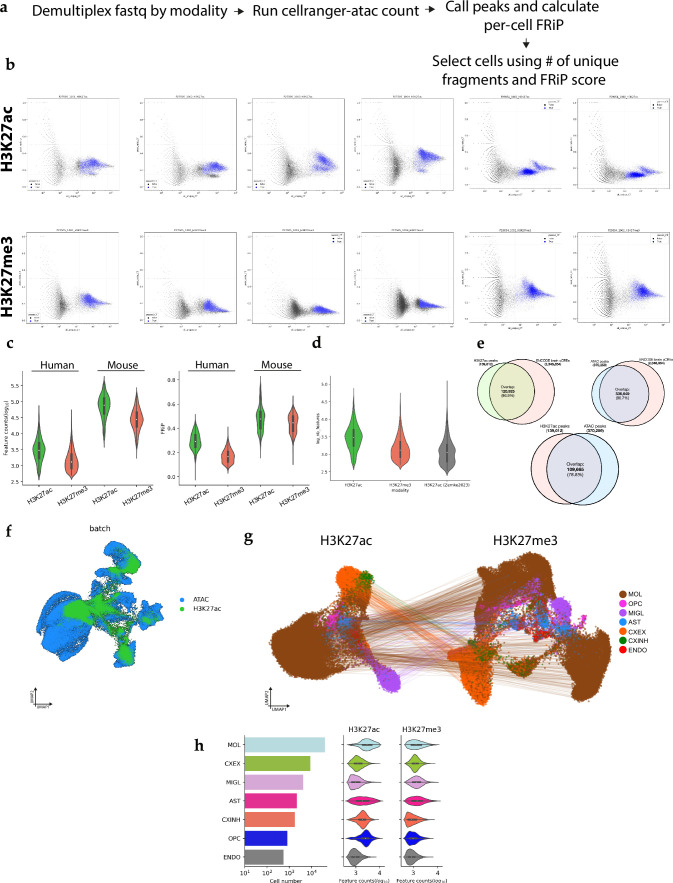
Quality assessment of H3K27ac/H3K27me3 nanoCUT&Tag. **a**, Workflow for demultiplexing and custom cell-calling for each nanoCUT&Tag antibody (H3K27ac, H3K27me3). **b**, Scatterplots used for custom cell calling in the H3K27ac (top) and H3K27me3 (bottom) modality. Number of unique fragments (log scale) shown on x axis and the fraction of reads in peaks (FRiP) shown on y axis. Selected cells shown in blue. **c**, Quality metric violin plots showing the number of unique features (left) and fraction of reads in peaks (FRiP) for H3K27ac (green) and H3K27me3 (red) in this dataset compared to those in a previously published dataset in mouse^29^. Black box within each violin plot represents the interquartile range (IQR), white dot is the median, and whiskers extend to 1.5× IQR. **d**, Violin plot showing the number of unique features (log scale) of the H3K27ac, H3K27me3 (this study) compared to the H3K27ac of a published dataset^33^. Black box within each violin plot represents the interquartile range (IQR), white dot is the median, and whiskers extend to 1.5× IQR. **e**, Venn diagrams showing the overlap between peaks called in our snATAC-seq dataset (blue), H3K27ac dataset (green) and a set of brain cCREs from the ENCODE database (pink). **f**, 2D UMAP co-embedding of the snATAC and H3K27ac nanoCUT&Tag datasets. **g**, 2D UMAP of the nanoCUT&Tag dataset from both modifications. Colored lines connect the same cell in both modalities. **h**, Bar chart and violin plot showing the cell number in each population (left) and distribution of feature counts (log scale) for each modality (middle, right). Data from each plot are composed of total cell counts for individual cell types. Black box within each violin plot represents the interquartile range (IQR), white dot is the median, and whiskers extend to 1.5× IQR.

Histone PTMs provide key functional information about local chromatin state^17,40–42^. We adapted our recently developed nanoCUT&Tag^29^ for archival human tissue (Methods) and simultaneously mapped H3K27ac (active mark) and H3K27me3 (repressive mark) in three CSCs and three cortical samples from four donors (Fig. 1a,d). Custom nano-Tn5 barcodes enabled demultiplexing of the two modalities, while the shared cell barcode linked each modality to the same cell, yielding 66,113 and 66,727 barcodes for H3K27ac and H3K27me3, respectively, and with 58,696 shared cells bearing both marks^29^ (Extended Data Fig. 4a,b and Methods). We captured a median of 3,038 and 1,315 unique fragments for H3K27ac and H3K27me3, respectively, comparable to metrics in our published mouse datasets and higher than in a H3K27ac droplet Paired-Tag dataset in the human M1 cortex^29,33^ (Extended Data Fig. 4c). The fraction of reads in peaks was 0.29 and 0.17 for H3K27ac and H3K27me3, respectively, consistent with the expectations for the nanoCUT&Tag chemistry and with the quality of archival tissue (Extended Data Fig. 4d and Methods). Comparison of snATAC and H3K27ac peaks with ENCODE brain-specific candidate *cis*-regulatory elements (cCREs) showed strong concordance, with 86.9% overlap between H3K27ac and ENCODE, 90.7% overlap between snATAC and ENCODE and with 78.8% overlap between snATAC and H3K27ac (Extended Data Fig. 4e). In all cell types, most peaks were intronic (median = 49%) and intergenic (median = 26%), consistent with the expected location of regulatory elements.

**Extended Data Fig. 5:**
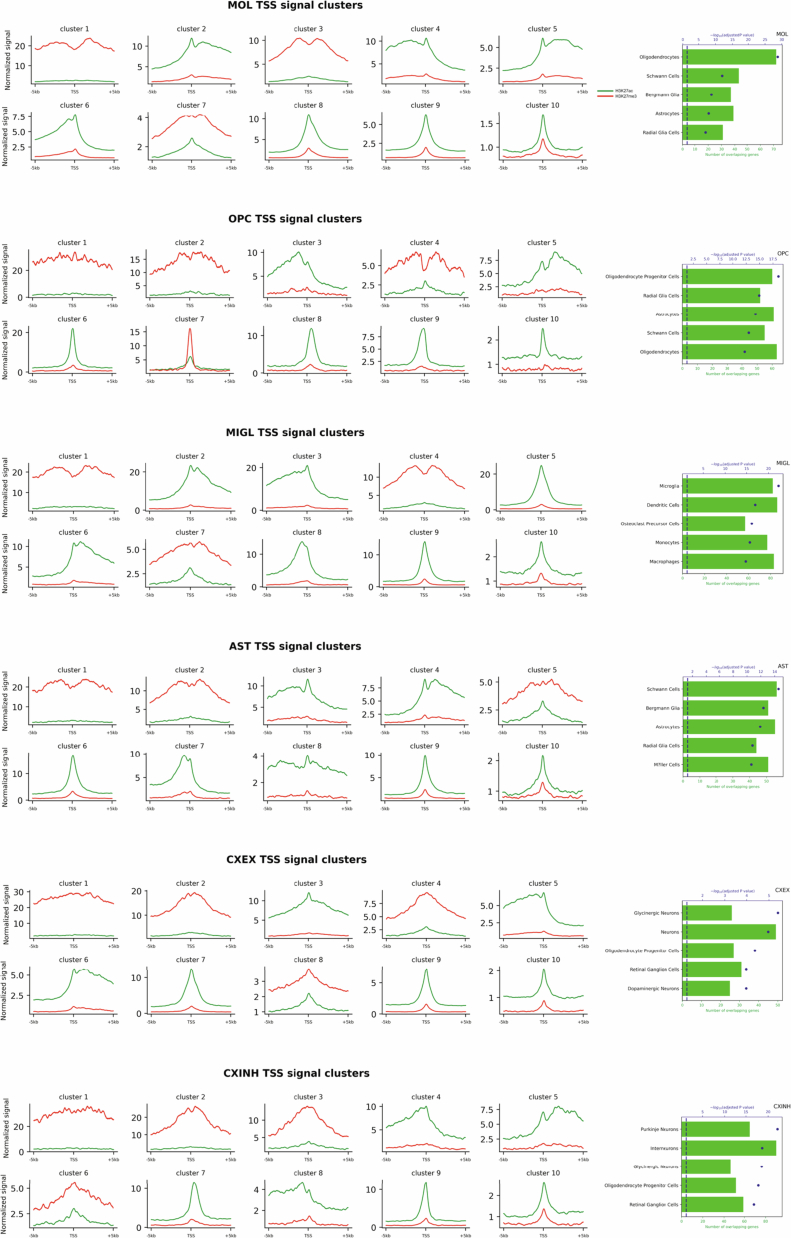
Cell-type-specific enrichment of H3K27ac/H3K27me3 across gene TSS. The k-means clustering of H3K27me3 and H3K27ac signal distribution at the TSS of all genes for each cell type. Genes in the clusters showing strong H3K27ac and weak H3K27me3 were used as input for gget enrichr analysis to identify enriched cell types. Top identified cell type is the cell type itself highlighting the strong cell-type-specific signal captured in our nanoCUT&Tag dataset.

H3K27ac marks active enhancers and promoters^40^, and correlates strongly with chromatin accessibility. We therefore integrated the H3K27ac and snATAC datasets (Extended Data Fig. 4f,g and Methods), and, after label transfer, we identified MOL (40,268 cells), CXEX (8,821 cells), MIGL (4,297 cells), AST (2,169 cells), CXINH (1,774 cells), OPC (820 cells) and ENDO (547 cells) populations in the H3K27ac dataset (Extended Data Fig. 4h). Genome browser tracks showed the expected enrichment of the respective signal for each of the identified cell types (Fig. 1f). To ascertain the quality of cell-type-specific histone PTM landscapes, we clustered genes according to their joint H3K27ac and H3K27me3 profiles in each cell type. This revealed distinct groups of genes, with increased active or repressive marks. We then performed a cell-type enrichment analysis on the genes in the active-mark-enriched clusters and confirmed that they were most strongly associated with the same cell types from which they were derived (Extended Data Fig. 5).

To assess antibody specificity, we generated meta-signal plots for both modalities using H3K27ac and H3K27me3 peaks in MOLs. Each mark showed strong enrichment at its corresponding peak set with minimal cross-mapping, confirming signal specificity^43^. We also verified robust H3K27ac enrichment and the absence of H3K27me3 at snATAC peaks, and in a published bulk H3K27ac dataset^32^ (Fig. 1g). As no comparable human brain H3K27me3 dataset exists for benchmarking, our data represent a unique resource providing both H3K27ac and H3K27me3 at single-cell resolution in the adult brain and spinal cord (SC).

We then used the ATAC, H3K27ac and H3K27me3 human CNS single-nuclei datasets to perform trimodal clustering of the entire genome and identify patterns and correlations among the three modalities in neural cell types (Fig. 1h and Methods). Although we identified the expected patterns of the three signals across the genome, the overall matrix looked scattered, highlighting not only the complexity of the regulatory genome but also the sparsity of the datasets. We identified different regions of the genome that were (1) accessible in all cell types, likely corresponding to housekeeping genes; (2) specifically accessible in each cell type with corresponding H3K27ac signal; and (3) presented shared H3K27me3 in all cell types (Fig. 1h). We then used the signal in all genomic bins to examine correlations among the different cell types, and observed, as expected, strong anticorrelation between the active ATAC and H3K27ac marks with the inactive H3K27me3 mark (Fig. 1i). Interestingly, chromatin accessibility exhibited a higher correlation across the different cell types overall, unlike H3K27ac, which presented higher correlation between glial populations or between neuronal populations (Fig. 1i). These findings suggest that H3K27ac may be a better discriminant of cell-type-specific regulatory activity compared to chromatin accessibility.

### Identification of a new enhancer for the *SOX10* gene in human OLGs

**Fig. 2:**
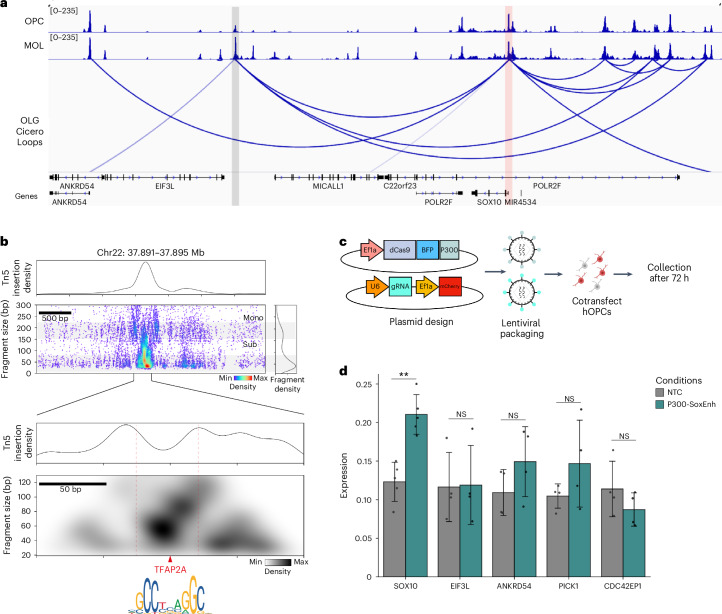
Characterization of a distal *SOX10* enhancer in the OLG lineage. **a**, snATAC–seq genome browser snapshot showing pseudobulk chromatin accessibility signal in OPCs and MOLs at the identified SOX10-distal enhancer (gray) and SOX10 locus (pink) and the corresponding loops identified using Cicero. **b**, The V plot showing tagmentation pattern and density in a 3.5-kb locus around the SOX10-distal enhancer. Top: density plot showing density of the Tn5 insertion events, seen enriched at the site of the enhancer. Bottom right: fragment size distribution, with subnucleosomal and mononucleosomal bands visible. Bottom left: scatterplot of fragments. Dots represent midpoints of fragments in the OLG lineage and colored by density of fragments. A 330-bp locus zoom-in at the enhancer site. TF footprint is seen as dip in the Tn5 insertion frequency (top) and is marked by a red dotted line. The V plot shows density of fragments at the center of the footprint. A TFAP2A motif was found in the center of the footprint. **c**, Schematic showing plasmid and lentivirus setup for CRISPRa experiment. **d**, Gene expression (qPCR) of five genes from CRISPRa experiment with guides targeting either the SOX10 enhancer (turquoise) or an NTC (gray). *n* = 4 biological replicates; data shown as mean ± s.e.m.; statistics, two-sided *t* test performed; *P ≤ 0.05, **P ≤ 0.01, ***P ≤ 0.001. NS, nonsignificant; NTC, nontargeting control. Schematic in **c** created in BioRender; Castelo-Branco, G. https://biorender.com/3ekzpp9 (2025). Source data

**Extended Data Fig. 6:**
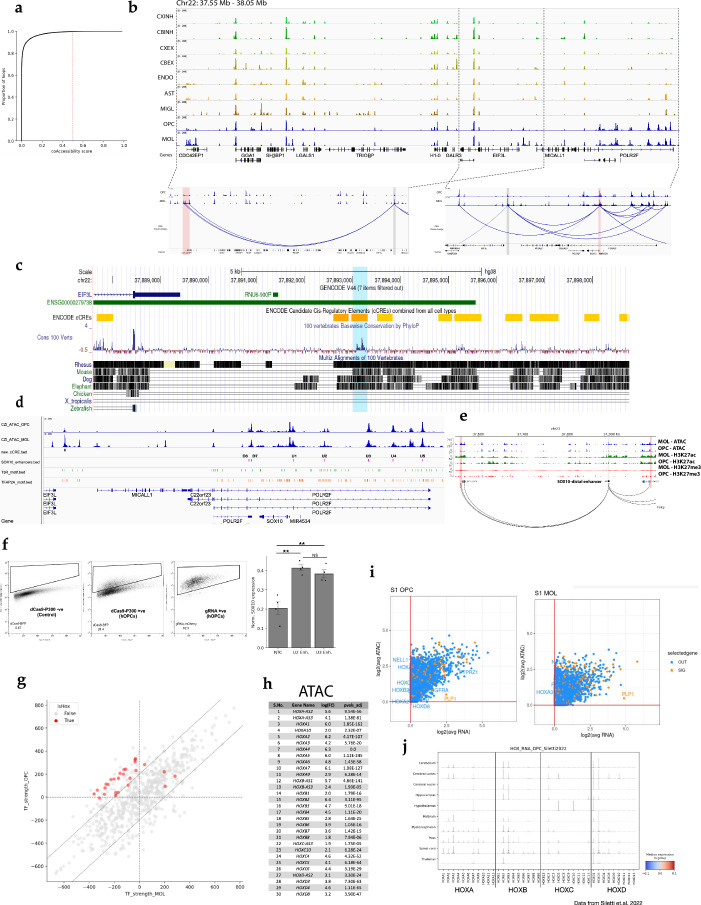
Co-accessibility analysis identifies a distal *SOX10* enhancer in OLGs. **a**, Cumulative distribution of the co-accessibility score for all loops identified by Cicero. Red line shows the score cutoff (0.5) used for assessing high-quality interactions and captures the top 5% of all loops. **b**, snATAC-seq genome browser snapshot showing chromatin accessibility signal in all cell types at *CDC42EP1* locus (left), identified SOX10-distal enhancer (middle) and *SOX10* locus (right) and the corresponding loops identified using Cicero. Red columns highlight the *CDC42EP1* and *SOX10* genes, and the gray column highlights the new enhancer. **c**, UCSC Genome Browser snapshot showing the identified enhancer locus (light blue column) as well as overlap with previously identified ENCODE cCREs, PhyloP base conservation score and evolutionary conservation with different species. **d**, snATAC-seq genome browser track showing chromatin accessibility in OPCs and MOLs at the newly identified enhancer, SOX10 locus, and previously characterized U1-U5, D6 and D7 enhancers (purple) and overlap with thyroid hormone receptor motifs (T3R, green) and TFAP2A motifs (orange). **e**, snATAC-seq and nanoCUT&Tag genome browser tracks the CDC42EP1-enhancer-SOX10 locus showing ATAC, H3K27ac and H3K27me3 pseudobulk signal in MOLs and OPCs and Cicero links. **f**, FACS plots showing transduction efficiency (left) and gene expression changes (qPCR) of *SOX10* when directing dCas9–p300 to the U2 enhancer, U3 enhancer, and a nontargeting control (NTC) (right). N = 4 biological replicates; data shown as mean ± s.e.m. Statistics: one-way ANOVA with Tukey’s post hoc test. Two-sided t-test performed. *p ≤ 0.05, **p ≤ 0.01, ***p ≤ 0.001, NS = nonsignificant. **g**, Scatterplot showing TF strength of shared core TFs in MOLs (x axis) and OPCs (y axis) and highlighting the identified HOX genes. **h**, List of identified HOX genes being differentially accessible in spinal cord OPCs and MOLs. **i**, Scatterplot showing correlation between the multiome-RNA and multiome-ATAC. **j**, Stacked violin plot showing gene expression levels of all HOX genes in OPCs in all regions from an adult human brain transcriptomic atlas^4^. Source data

Promoters and enhancers exhibit open chromatin and H3K27ac enrichment, making our datasets well-suited to identify cCREs (Extended Data Fig. 6a,b, Supplementary Table 2 and Methods). To validate this, we focused on the *SOX10* gene, which has several well-characterized enhancers^44^. Indeed, the *SOX10* promoter was co-accessible with peaks corresponding to the enhancers in OLG, but not other lineages^45^ (Extended Data Fig. 6a,b). Notably, we found that the promoter was also co-accessible with two distal upstream peaks, previously not associated with *SOX10* (Fig. 2a and Extended Data Fig. 6b). One peak corresponded to the *ANKRD54* gene promoter, which we found accessible in all cell types. However, the second peak, located 91 kb upstream of *SOX10*, was accessible only in the OLG lineage, suggesting it may be an uncharacterized OLG-specific enhancer.

Interestingly, this distal enhancer was co-accessible with the *CDC42EP1* promoter, located 300-kb away (Extended Data Fig. 6b). *CDC42EP1* encodes the effector protein of CDC42, which is associated with myelin sheath compaction in MOLs^46^. Notably, we observed increased accessibility of the *CDC42EP1* promoter specifically within MOLs, but not OPCs (Extended Data Fig. 6b–d). We checked the co-accessibility links within the OPCs and MOLs separately, and found *SOX10* interactions with the canonical enhancers, and the new enhancers in both populations, but the *CDC42EP1* promoter–enhancer connection only in MOLs (Supplementary Table 2), suggesting that the chromatin looping concerning this enhancer is altered upon OLG lineage progression. We also queried this locus in our nanoCUT&Tag dataset and observed an increase in the H3K27ac signal at the identified enhancer in MOLs and OPCs, with an increase in H3K27ac at the *CDC42EP1* promoter in MOLs specifically (Extended Data Fig. 6e).

This enhancer was evolutionarily conserved across four species, including rhesus monkey and mouse (Extended Data Fig. 6c), and displayed positive PhyloP scores^47^, characteristic of enhancer evolution^48^. Using volcano (V) plots^49,50^ in a 3.5-kb locus spanning the enhancer, we found a strong density of fragments corresponding to the subnucleosomal and mononucleosomal bands, but also intermediate-length fragments, indicative of dynamic TF-bound open chromatin (Fig. 2b). A focused 300-bp window revealed a clear TF footprint in the tagmentation density, corroborated by the expected fragment distribution in the V plot (Fig. 2b). A motif analysis of the core footprint identified a TFAP2A motif coinciding well with the expected binding site from the V plot. The AP2-α TF has been shown to regulate *SOX10* expression, but specifically through the U3 enhancer^44^, which is distinct from this distal enhancer. This suggests that a new distal enhancer may be regulating *SOX10* expression in the OLG lineage.

To test enhancer function, we performed a CRISPR activation (CRISPRa) assay in induced pluripotent stem cell (iPSC)-derived human OPCs^51,52^, using dead-Cas9 fused to the transcriptional co-activator p300 (dCas9–p300 (ref. ^53^); Fig. 2c). Recruiting dCas9–p300 to the enhancer element increased *SOX10* expression but not neighboring genes *EIF3L*, *ANKRD54*, and *PICK1* or *CDC42EP1* (Fig. 2d). Collectively, these findings propose that, in addition to U2 and U3 (Extended Data Fig. 6f), this new distal enhancer can regulate *SOX10* in the human OPCs.

### Core regulatory TF networks in adult human neural cell types

**Fig. 3:**
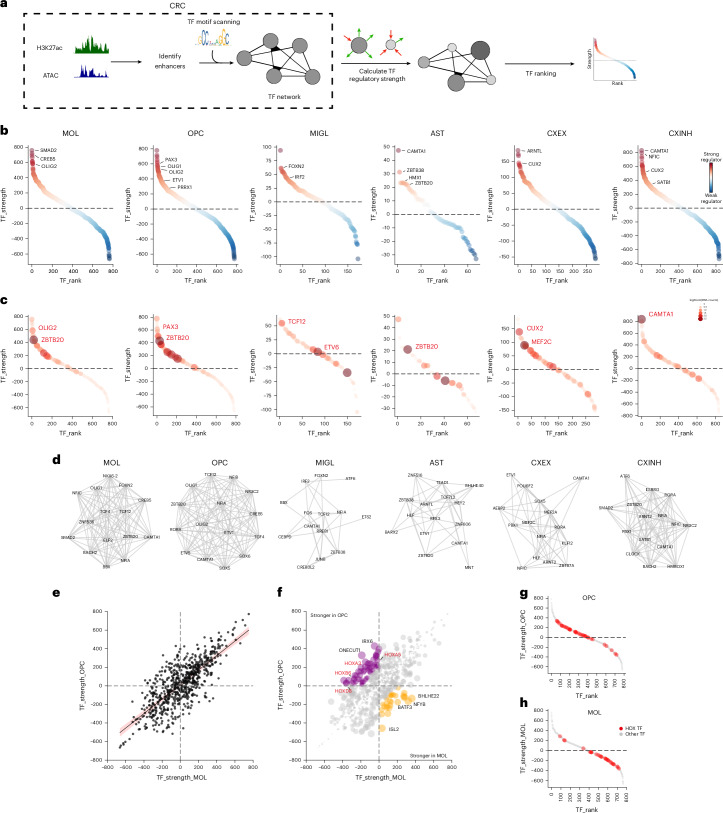
Core TF networks reveal lineage-specific regulators. **a**, Schematic for constructing the regulatory TF circuit and assigning a regulatory strength based on the difference in the incoming and outgoing connections for each TF node in the network. **b**, Ranked scatterplot showing the strength of core TFs identified within the regulatory network for different cell types. Strength is measured by the difference in number of outgoing connections (out degree) and incoming connections (in degree). Top TFs are marked for each population. **c**, Same plot as in **a**, but size and color intensity of each dot represents the average (log-normalized) expression of that TF in the population. The TF with the highest expression in each network is shown in red. **d**, Network of the top 15 TFs (by expression) in each cell type. Density of edges in the network reflects the correlation between the TF strength and the expression of the TF. **e**, Scatterplot showing the strength of the shared TFs in the OPC and MOL core networks. Red line represents linear regression line, with 95% confidence interval shown as shaded region. **f**, Same as in **e**, but size of the dots represents the difference in the strength of the TF in the OPC and MOL populations. TFs stronger in OPC and MOL are shown in purple and orange, respectively. A subset of the HOX TFs is marked in red. **g**, Ranked scatterplot highlighting the position of the HOX TFs in the OPC population. **h**, Same as **g**, but in MOL population. CRC, core regulatory circuit.

Using the two datasets, we constructed a core TF regulatory network for each identified cell type based on enhancer and TF motif accessibility (Fig. 3a and Methods). Alongside expected TFs such as OLIG2 in MOLs and OPCs, IRF2 in MIGL and CUX2 and SATB1 in cortical neurons, we identified new regulators, including FOXN2 in MIGL and ZBTB20 and HMX1 in AST (Fig. 3b and Supplementary Table 3). To assess FOXN2 specificity in microglia, we compared it with other forkhead box (FOX) TFs in the network—FOXK1, FOXO3, FOXI1, FOXJ1, FOXJ2 and FOXP3. Although FOXK1 and FOXO3 showed similar expression levels to FOXN2, both had lower regulatory strength. FOXK1 has been implicated in the regulation of STAT1 expression in microglia^54^, and FOXO3 has well-characterized roles in microglial responses to neuroinflammation and phagocytosis^55,56^, suggesting that FOXN2 may have a distinct regulatory role in this lineage. In OPCs, we identified PAX3 potentially reflecting a dorsal lineage contribution^11^, and PRRX1, a regulator of OPC quiescence^57^, which was absent in MOLs. Notably, with the exception of CAMTA1 in CXINH, TF expression level did not necessarily predict regulatory strength; ZBTB20, which is upregulated after cerebral ischemia in neural progenitor cells^58^, was the highest expressed TF in the AST, MOL and OPC populations but ranked only 9th, 20th and 29th in regulatory strength, respectively (Fig. 3c,d).

We also investigated how TF networks change during OLG differentiation by comparing OPCs and MOLs. Over 90% TFs in the networks were shared among the two populations and showed comparable regulatory strength (Fig. 2e). However, each population also contained distinct high-strength regulators that may support lineage-specific functions, including ARX, MYC, SIX1 and FOXF2 in OPCs, and NFAT5, ARNT, ZNF566 and ZNF333 in MOLs (Supplementary Table 3).

We then examined TFs whose regulatory strength changed across OLG differentiation and found, among others, BHLHE22 as a strong TF in the MOLs (Fig. 3f). *BHLHE22* expression has been shown to increase in OPCs upon T3 stimulation and has a role in differentiation and myelination^59^. In OPCs, ONECUT1, which regulates *NKX6-2* expression, a key TF in OPCs, showed stronger activity. Interestingly, we found several HOX TFs, including HOXA3, HOXA5 and HOXB6, as strong regulators in OPCs, but weaker in MOLs (Fig. 3f and Extended Data Fig. 6g), suggesting higher regulatory potential in progenitors that diminishes with differentiation (Fig. 3g,h). The HOX TFs appeared only in the OLG lineage and not in other cell types, indicating a lineage-specific regulatory role (Supplementary Table 3).

### SC adult OLGs exhibit increased accessibility at HOX family genes, decoupled from gene expression

**Fig. 4:**
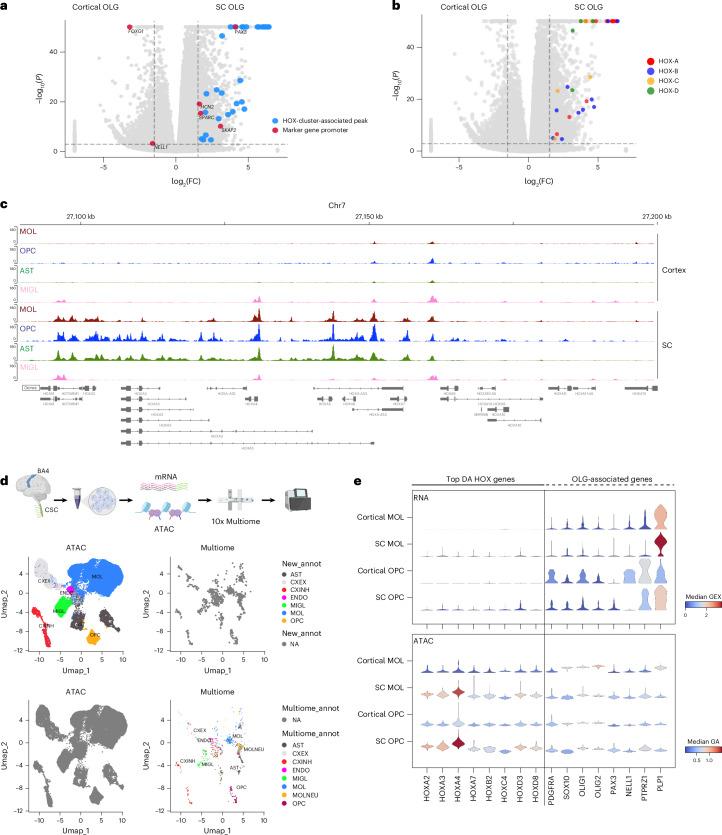
Region-specific accessibility highlights HOX enrichment in SC OLGs. **a**, Volcano plot showing differentially accessible peaks in SC and cortical OLGs. Previously characterized marker genes are shown in red and labeled. HOX cluster-associated peaks are shown in blue. Two-sided *t* test with Benjamini–Hochberg correction. Thresholds, adjusted *P* = 0.001, log(FC) = 1.5. **b**, Same as in **a**, but highlighting the specific HOX clusters identified as being differentially accessible. Two-sided *t* test with Benjamini–Hochberg correction. **c**, snATAC–seq genome browser snapshot showing pseudobulk chromatin accessibility signal in AST, MIGL, MOL and OPC populations from the CSC and motor cortex at the *HOXA* locus. MIGL signal is depleted in both regions, whereas AST, OPC and MOL exhibit accessibility in SC. **d**, Schematic showing workflow for the multiome experiment and UMAP embedding of the multiome ATAC with cell types annotated using the multiome-RNA. **e**, Stacked violin plots showing the expression (top) and promoter accessibility (bottom) in cortex and SC-derived MOLs and OPCs from a multiome experiment. Most differentially accessible HOX genes and OLG marker genes are shown. Schematic in **d** created in BioRender; Castelo-Branco, G. https://biorender.com/orfbpyi (2025).

The HOX family of proteins comprises evolutionarily conserved TFs that have key roles in the developmental patterning of the embryonic SC^60^. As we observed potential regulatory activity in adult OLGs, we asked whether there was regional specificity. Differential accessibility analysis between motor cortex (BA4) and CSC-derived OLGs revealed regionally enriched genes previously reported by snRNA-seq, such as *PAX3*, *SKAP2*, *SPARC*, *HCN2* in CSC OLGs and *FOXG1*, *NELL1* in BA4-OLGs^5^ (Fig. 4a). Notably, 30 HOX cluster genes showed higher accessibility in CSC OLGs than in cortical OLGs (Fig. 4a,b and Extended Data Fig. 6g). Genome browser tracks showed that the OLG lineage and AST, but not MIGL, presented increased HOX accessibility in the SC (Fig. 4c), matching the developmental HOX expression pattern of the cervical spinal cord^61^.

As promoter accessibility can reflect transcriptional activity, we coprofiled chromatin accessibility and RNA using the 10x Genomics multiome platform on cervical spinal cord and cortical samples (Fig. 4d). Integration with the snATAC dataset confirmed strong concordance between accessibility and expression of core OLG genes, such as *PTPRZ1* (OPCs), *PLP1* (MOLs) and the TF-encoding *SOX10*, *OLIG1*, *OLIG2* genes were highly correlated (Fig. 4e). In contrast, eight HOX genes showed elevated accessibility in CSC OLGs, but only residual RNA expression (Fig. 4e). To eliminate sequencing depth as a factor, we analyzed HOX expression in a high-depth single-cell transcriptomic atlas^4^ of the adult human brain, which revealed similar residual HOX gene expression (Extended Data Fig. 6j), indicating a transcriptional and epigenomic decoupling at HOX loci in adult OLGs, unlike during development.

### *HOXA*/*HOXD* genes are primed for expression in subsets of SC-derived adult human OLG

**Fig. 5:**
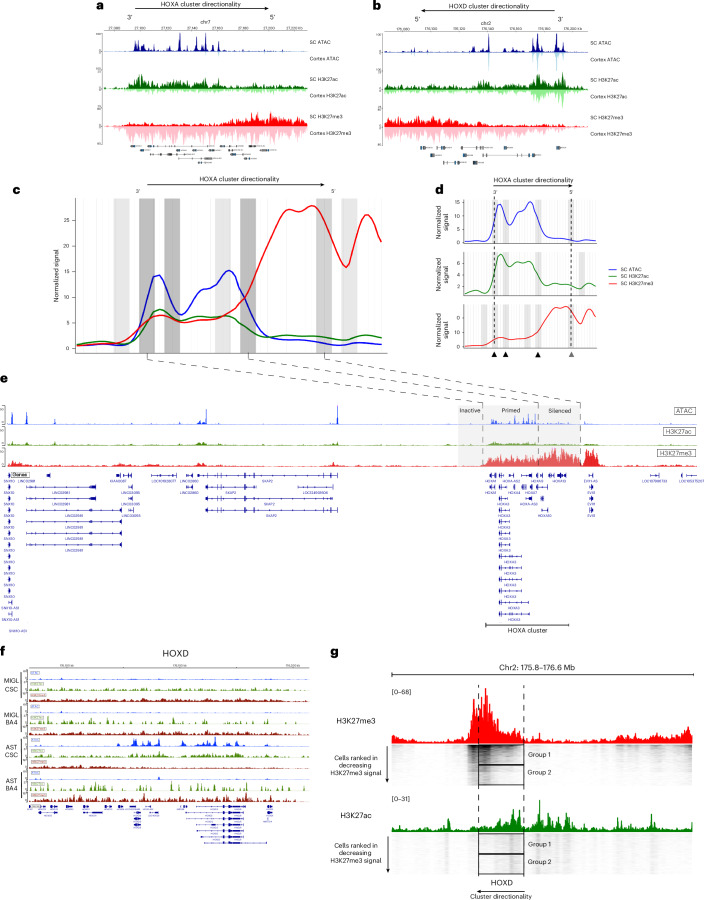
Adult SC OLGs show developmental HOX chromatin domains. **a**, nanoCUT&Tag and snATAC–seq genome browser tracks showing H3K27ac, H3K27me3 and ATAC pseudobulk signals in OLGs at the HOXA in CSC (upright track, darker shade) and motor cortex (inverted track, lighter shade). **b**, Same as in **a**, but for the *HOXD* locus. **c**, Gaussian smoothed normalized signal from ATAC (blue), H3K27ac (green) and H3K27me3 (red) across the HOXA cluster with a 50-kb flanking region upstream and downstream. Gray bars show the locations of the cumulative ‘signal boundaries’ identified in each modality. Color intensity reflects the cumulative strength of the signal boundary. **d**, Same as in **c**, but with each modality separated out. HOXA directionality is shown at the top, and arrows beneath show the medium (two modalities) and strong (three modalities) signal boundaries. **e**, nanoCUT&Tag and snATAC–seq genome browser track of the HOXA cluster showing the location of the strong signal boundaries and the corresponding inactive, primed and silenced chromatin domains. **f**, nanoCUT&Tag and snATAC–seq genome browser tracks showing the ATAC (blue), H3K27ac (green) and H3K27me3 (red) pseudobulk signal in the microglial and astrocyte populations at HOXD in both SC (CSC) and cortex (BA4). **g**, nanoCUT&Tag genome browser track around the *HOXD* locus (marked with dotted lines) with H3K27me3 (red) and H3K27ac (green) pseudobulk signal in SC OLGs. Single-cell tracks are shown below and sorted by decreasing H3K27me3 signal. Group 1 cells exhibit moderate H3K27me3 at the 3′ end, while group 2 cells show H3K27me3 depletion, and the amount of H3K27ac remains the same in both groups, suggesting that group 2 cells may be expressing low levels of HOX genes.

**Extended Data Fig. 7:**
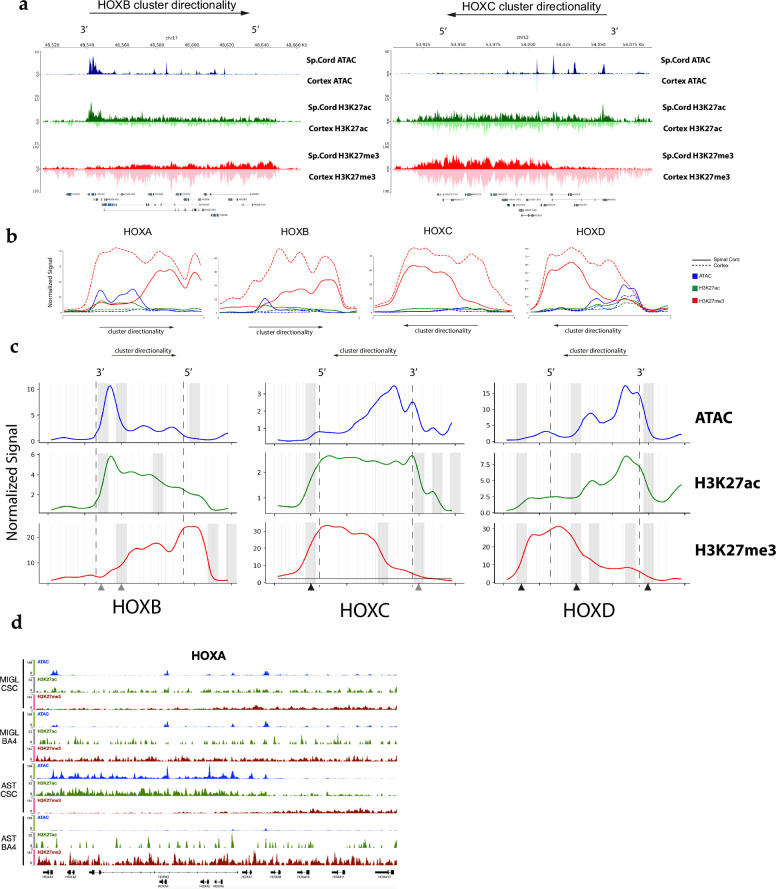
Chromatin accessibility and histone PTM patterns at HOX loci in OLGs. **a**, snATAC-seq and nanoCUT&Tag genome browser tracks showing H3K27ac, H3K27me3 and ATAC pseudobulk signal in OLGs at the HOX-B and HOX-C clusters in cervical spinal cord (upright track, darker shade) and motor cortex (inverted track, lighter shade). Directionality of the clusters is shown by the arrow. **b**, Gaussian smoothed normalized signal from ATAC (blue), H3K27ac (green) and H3K27me3 (red) in spinal cord OLGs (solid line) and cortical OLGs (dotted line) across each HOX cluster with a 50 kb flanking region upstream and downstream. **c**, Gaussian smoothed normalized signal from ATAC (blue), H3K27ac (green) and H3K27me3 (red) across the HOXB, HOXC and HOXD clusters with a 50 kb flanking region upstream and downstream, separated by each modality. Gray bars show the location of ‘signal boundaries’ identified in each modality. Dotted lines mark the boundaries of each HOX cluster. Arrowheads underneath the plots mark intermediate and strong signal boundaries. HOX cluster directionality shown by arrow on top. **d**, snATAC-seq and nanoCUT&Tag genome browser tracks showing the ATAC (blue), H3K27ac (green), and H3K27me3 (red) pseudobulk signal in the microglial and astrocyte populations at HOXA in both spinal cord (CSC) and cortex (BA4).

Because HOX genes present open chromatin but attenuated expression in adult human OLGs, we examined H3K27ac and H3K27me3 deposition in SC OLGs and cortical OLGs at these loci. Cortical cells displayed a uniform H3K27me3 across the clusters, reflecting the canonical pattern of Polycomb Repressive Complex 2 (PRC2) mediated repression^62^ (Fig. 5a,b and Extended Data Fig. 7a,b). In contrast, H3K27me3 and H3K27ac exhibited anticorrelated gradients across the *HOXA–HOXD* clusters in SC OLGs (Fig. 5a,b), reminiscent of *HOX* gene collinearity during development^61–63^. The 3′ ends of the clusters (for example, *HOXA1* to *HOXA7*) showed elevated ATAC, H3K27ac with reduced H3K27me3, while the 5′ ends (for example, *HOXA10* to *HOXA13*) showed the opposite, with a sharp transition in the middle. This pattern matches the developmental CSC HOX activation profile. Chromatin accessibility was also lower in differentiated postmitotic MOLs relative to OPCs (Fig. 4c), supporting the idea that OPCs retain epigenetic memory of developmental chromatin states, potentially through mitotic inheritance.

To assess domain organization across the HOX clusters, we compared signal transitions across chromatin accessibility, H3K27ac and H3K27me3, which revealed multiple discrete domains with coordinated changes (Methods). Within the HOXA cluster, we identified three strong, one moderate and one weak border (Fig. 5c,d). Two strong borders flanked *HOXA1* to *HOXA4* and were present in all modalities (Fig. 5c–e). A weak border around *HOXA7* marked a region with increased H3K27me3 and increased accessibility, while a strong border at *HOXA10* corresponded to the shift from predominantly accessible to predominantly H3K27me3-repressed chromatin. A moderate border marked the decrease of the heavily inactive signature at the 5′ end of the cluster (after *HOXA13*). The strong border identified at the 3′ end of the cluster suggested further nuance. Although the levels of H3K27me3 at the 3′ end (*HOXA1* to *HOXA7*) were far lower than the 5′ end, it was distinctly greater than the flanking chromatin immediately upstream of the cluster and to the left of the identified border. Thus, we could demarcate the following three regulatory domains around the HOXA cluster: (1) inactive chromatin upstream of the cluster, (2) primed chromatin at the 3′ end and (3) silenced chromatin at the 5′ end (Fig. 5e). While the *HOXB* and *HOXC* clusters displayed moderate borders at the 3′ end of the respective clusters, the *HOXD* cluster displayed strong borders at the 3′ end and in the middle of the cluster, as in the *HOXA* cluster (Extended Data Fig. 7c). Thus, this indicates three distinct levels of the H3K27me3 repressive mark at HOX loci in adult OLGs, suggesting that this increased level of H3K727me3 at the 3′ end might be sufficient to prevent gene expression and maintain the genes in a primed state. In line with the accessibility pattern seen in SC astrocytes earlier, we also found a similar H3K27ac and H3K27me3 pattern within this population that was absent in cortical astrocytes and in both SC and cortical microglia (Fig. 5f and Extended Data Fig. 7d), suggesting that these priming mechanisms could also be operational in astrocytes.

The multimodal nanoCUT&Tag data enabled joint analysis of H3K27ac and H3K27me3 within the same cell. In the HOXD cluster, all cells showed high H3K27me3 at the 5′ end (*HOXD8*–*HOXD13*), but the 3′ end displayed two groups with either medium (group 1) or low (group 2) H3K27me3. In contrast, H3K27ac at the 3′ end was uniform across all cells (Fig. 5g). This suggests that, while most adult OLGs retain a primed repressive state at the HOX loci, a subset of OLGs lacking H3K27me3 but with enriched H3K27ac may be capable of low-level HOX expression and could explain the low RNA expression seen in single-cell transcriptomic studies. Definitive confirmation would require simultaneous single-cell coprofiling of H3K27ac, H3K27me3 and RNA.

### Developmental architecture of HOX genes in iPSC-derived human OLGs

**Fig. 6:**
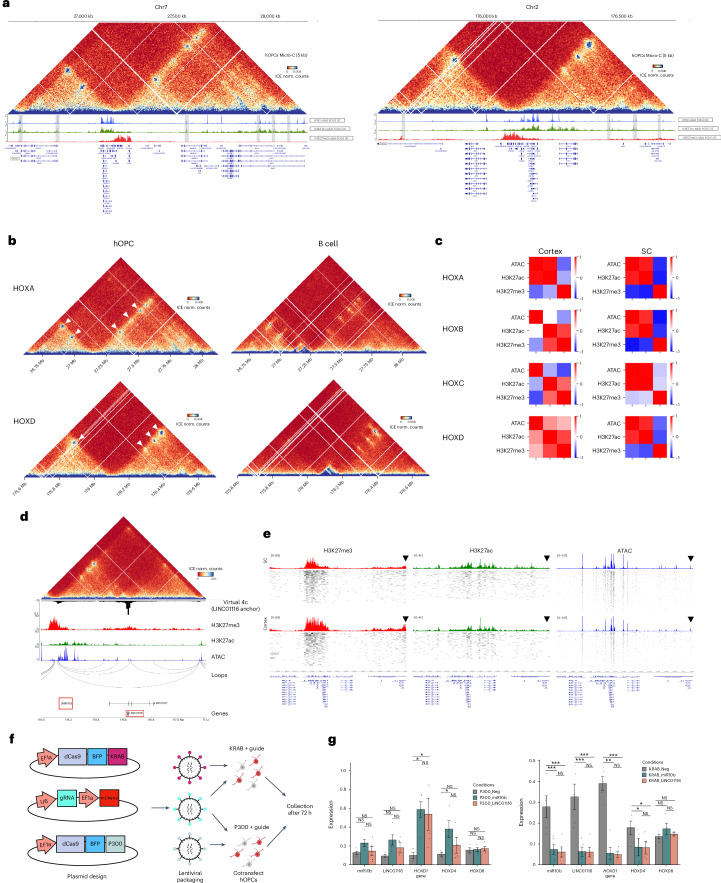
The 3D chromatin architecture in human iPS-derived OPCs recapitulates the developmental organization of HOX domains. **a**, Normalized Micro-C contact matrix at 5-kb resolution at *HOXA* (left) and *HOXD* (right) loci and corresponding ATAC, H3K27ac and H3K27me3 tracks in human adult SC OLGs showing the c-Dom and t-Dom TAD structures, including the sub-TAD contacts. Contacts with distal enhancers is shown by the gray bars. **b**, Micro-C contact matrix showing contacts between HOX genes and flanking enhancers in hOPCs, in contrast to B cells. **c**, Correlation matrix of ATAC, H3K27ac and H3K27me3 signals in cortex and SC-derived OLGs across all four HOX clusters. **d**, Contact matrix showing long-range interaction between miR10b and LINC01116, virtual 4c (anchored on LINC01116) H3K27me3, H3K27ac, ATAC and inferred loops are shown. **e**, scATAC, H3K27ac and H3K27me3 tracks showing signal distribution at HOXD and distal LINC01116 in SC OLGs and cortex OLGs. **f**, Schematic showing plasmid and lentivirus setup for CRISPRi/a experiment. **g**, Gene expression changes (qPCR) of five genes from the CRISPRi/a experiment. Left, data from CRISPRa (left, dCas9–p300) and CRISPRi (right, dCas9–KRAB) targeting either miR10b (turquoise), LINC01116 (red) or an NTC (gray). *n* = 4 biological replicates; data shown as mean ± s.e.m.; statistics, one-way ANOVA with Tukey’s post hoc test; two-sided *t* test performed; *P ≤ 0.05, **P ≤ 0.01, ***P ≤ 0.001. ANOVA, analysis of variance. Schematic in **f** created in BioRender; Castelo-Branco, G. https://biorender.com/orfbpyi (2025). Source data

**Extended Data Fig. 8:**
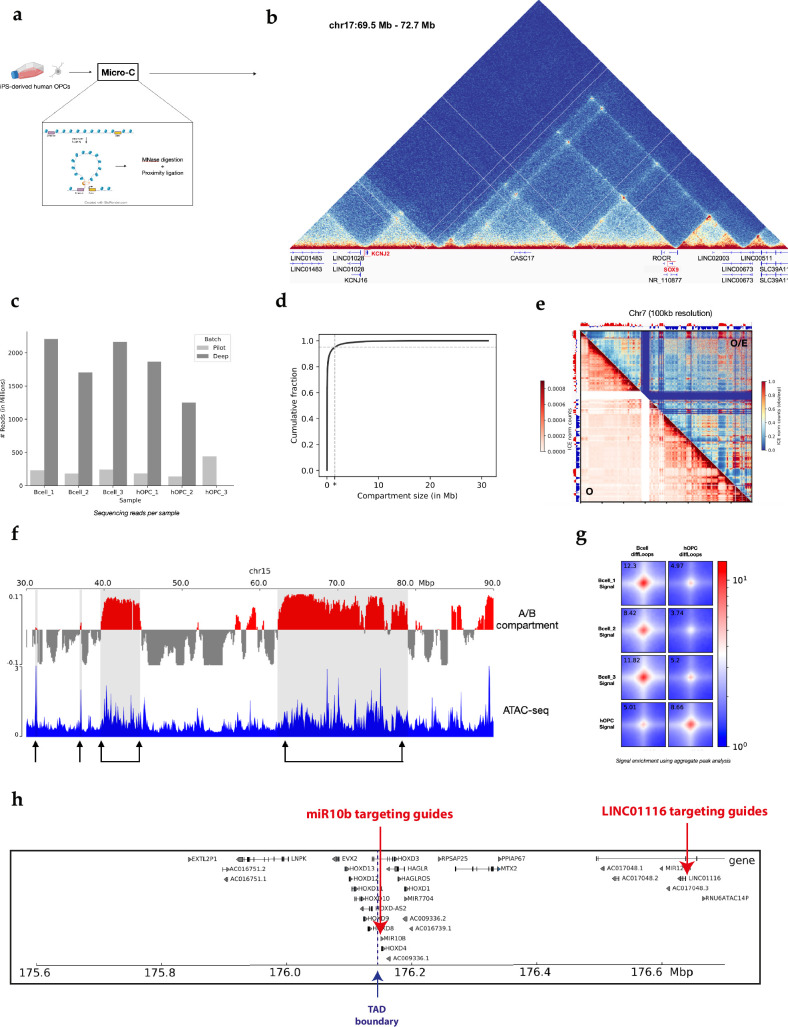
High-resolution Micro-C in human iPS-derived OPCs and primary B-cells shows cell-type-specific architecture. **a**, Experimental schematic showing collection of iPS-derived hOPCs and patient-derived B cells for Micro-C. Schematic of chromatin looping to bring enhancer and promoter in contact for transcription is shown below. Schematic in **a** created in BioRender. Castelo-branco, G. https://BioRender.com/orfbpyi (2025). **b**, Micro-C contact matrix in hOPCs at 5 kb resolution showing the TAD formed at the *SOX9–KCNJ*2 locus. **c**, Bar chart showing the sequencing reads (in millions) obtained for each replicate in the B cells and hOPCs. Libraries were first shallow sequenced to assess library quality (light gray bars) followed by deep sequencing (dark gray). B cell replicates correspond to 3 separate patients. hOPC replicates correspond to separate biological replicates. **d**, Cumulative distribution of compartment size identified in the hOPC and B-cell Micro-C data. Asterisk marks 1.5 Mb size as the upper size limit for 95% of all compartments. **e**, Contact matrix showing the normalized observed counts (lower triangle) and normalized obs/exp counts (upper triangle) at chromosome 7 in hOPCs. A and B compartments are shown along the sides of the matrix and exhibit strong correlation with ‘pockets’ of increased contact frequency. **f**, A/B compartments in hOPCs on chromosome 15 overlaid on chromatin accessibility data from hOPCs (unpublished), showing correlation between active A compartments and increased accessibility. **g**, Aggregate pileup analysis of differentially accessible loops in B cells and hOPCs showing cell type specificity of identified loops. **h**, Schematic of the 3’ TAD (c-Dom) and 5’ TAD (t-Dom) that flank the HOXD cluster (yellow) and the locations of genes tested in qPCR (*HOXD1*, *HOXD4*, *HOXD8*) and the *miR10b* and *LINC01116* genes.

HOX gene expression is shaped by chromatin architecture during development^62,63^. Given the observed epigenetic memory of the developmental state of chromatin at the HOX genes in adult OLGs, we questioned whether the 3D chromatin conformation has a role in the epigenetic state of HOX genes in OLGs. During embryogenesis, HOX activation involves dissolution of a single topologically associated domain (TAD) and formation of two domains (c-Dom and t-Dom) that connect HOX genes to flanking enhancers^61,63^. To examine the TAD structure in OLGs, we generated high-resolution Micro-C maps^64^ in iPSC-derived human OPCs^51,52^ and in human primary B cells to compare them with those of another developmental lineage (Fig. 6a and Extended Data Fig. 8a–e). We were able to capture broad compartment-level information^65^ and TAD structures^66^ at a resolution of 5 kb, which corresponded with the well-characterized *SOX9–KCNJ2* locus (Extended Data Fig. 8b). The active A compartments^65^ identified in the OPCs also corresponded to regions of high accessibility in OPCs, further strengthening the validity of the data (Extended Data Fig. 8f). Finally, we could also identify cell-type-specific loops and interactions in both B cells and the human OPCs (Extended Data Fig. 8g).

Around the *HOXA* and *HOXD* clusters, we observed distinct differences between hOPCs and B cells (Fig. 6b,c). In B cells, each cluster was sequestered into a single insulated TAD. In OPCs, however, each cluster was split into two larger TADs, with the 3′ and 5′ ends of the cluster engaging in interactions with distinct upstream and downstream regions (Fig. 6b,c), reminiscent of the developmental c-Dom and t-Dom configuration during activation in the development^63^. Overlaying these data with accessibility and histone-mark profiles from adult SC OLGs revealed that the boundary among these domains aligned with the strong chromatin border separating primed from repressed HOX regions suggesting the 3′ and 5′ genes of each cluster might be associated with an active and silent TAD, respectively (Fig. 6a). Within the TADs, we also observed subcontacts between regions outside the cluster. Interestingly, within the *HOXA* active TAD, we observed contact with a region in the *SKAP2* locus, which contains a well-known enhancer regulating the expression of 3′ *HOXA* genes during development^67^ (Fig. 6a).

We then investigated whether the expression of these HOX genes might be regulated by putative distal cCREs within the TAD domains in hOPCs. We observed a long-range contact between the HOXD-embedded microRNA *MIR10B* and a distal enhancer within the long noncoding RNA *LINC01116* (Fig. 6d), a regulatory contact previously seen in astrocytes^68^. This enhancer displayed increased accessibility and H3K27ac in SC OLG (Fig. 6e), suggesting that it may be active in these cells. To functionally assess its role in *HOXD* gene regulation, we performed CRISPRi/a assays (Fig. 6f and Extended Data Fig. 8h). Targeting the enhancer with dCas9–KRAB repressed *MIR10B*, *HOXD1* and *HOXD4* gene expression, while activation with dCas9–p300 led to an increase in their expression (Fig. 6g). Notably, *HOXD8*, situated close to *MIR10B* but in the adjacent c-Dom, remained insulated from the effects of KRAB and p300 (Fig. 6g). These results suggest that both the histone modification landscape and 3D architecture at the HOX loci regulate their transcriptional state in OLGs.

### HOX genes with primed chromatin in SC OLG are activated in high-grade gliomas

**Extended Data Fig. 9:**
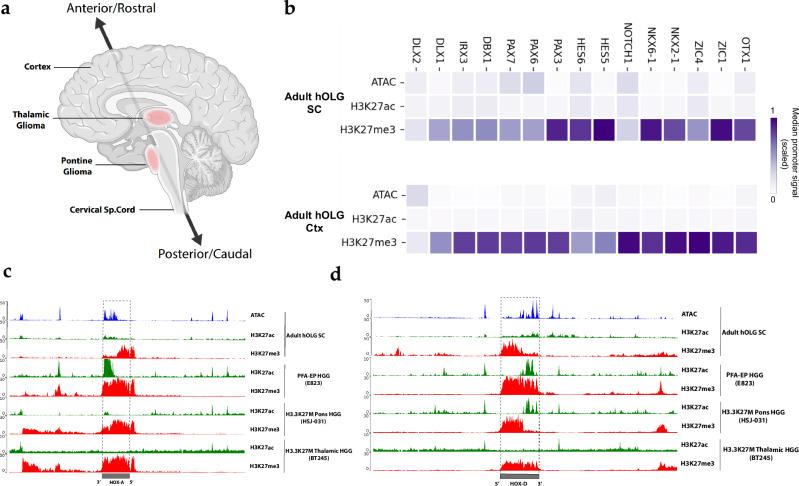
HOX-associated chromatin states in adult OLGs mirror regulatory programs in HGG. **a**, Schematic of the adult human brain showing the location of pontine and thalamic gliomas along the A-P axis. Schematic in **a** created in BioRender. Castelo-branco, G. https://BioRender.com/orfbpyi (2025). **b**, snATAC-seq and nanoCUT&Tag normalized promoter accessibility (ATAC), H3K27ac and H3K27me3 signal in spinal cord OLGs (top) and cortical OLGs (bottom) at different developmental genes associated with brain and spinal cord patterning. **c**, Genome browser tracks showing nanoCUT&Tag H3K27ac and H3K27me3 pseudobulk signal coverage at the HOXA cluster in spinal cord (SC) derived adult human OLG (hOLG) and PFA-EP tumors, H3.3K27M pontine tumors, and H3.3K27M thalamic tumors^11^. **d**, Same as **c**, but at the HOXD locus.

Ectopic activation of HOX genes is a feature of many cancers^61,69^. Midline H3K27M high-grade gliomas (HGGs) have been shown to have OPC origins^11,70^. These gliomas exhibit strong spatiotemporal specificity and disrupted PRC2 function^70,71^ (Extended Data Fig. 9a). Additionally, their HOX chromatin architecture faithfully reflects the developmental identity of their cell of origin^11^.

**Fig. 7:**
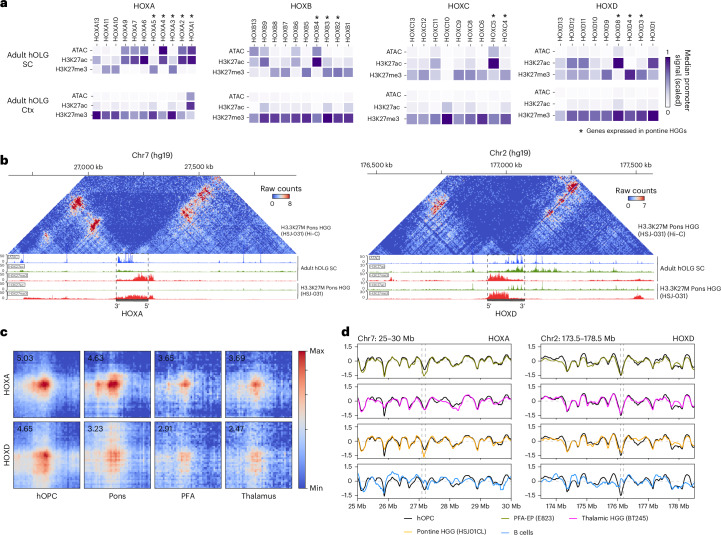
Primed HOX loci in adult OLGs mirror HOX activation in HGGs. **a**, nanoCUT&Tag and snATAC–seq normalized promoter accessibility (ATAC), H3K27ac and H3K27me3 signal in SC OLGs (top) and cortical OLGs (bottom) at all HOX genes. Asterisk indicates the genes previously identified to be expressed in pontine HGGs^11^. **b**, Normalized Hi–C contact matrix in H3.3K27M pontine HGG^11^ at the HOXA locus (marked by dotted lines) and corresponding ATAC, H3K27ac and H3K27me3 signals in SC OLGs and H3K27ac and H3K27me3 in H3.3K27M pontine HGG, showing similarity in mark distribution in nondiseased conditions and gliomas. **c**, Aggregate pileup analysis of hOPC loops (Micro-C) at HOXA (top) and HOXD (bottom) in pontine HGG, PFA-EP and thalamic HGG (left to right)^11^. **d**, Insulation score from the Micro-C matrix across a 5-Mb window spanning HOXA (left) and HOXD (right) in the three HGGs (green, pink, yellow, from ref. ^11^) and B cells (blue) overlaid on the hOPC insulation profile (black).

Given the primed accessibility, histone marks and 3D architecture we observed around HOX loci in SC OLGs, we asked whether this state relates to HOX activation in H3K27M gliomas. Several HOX genes activated in pontine HGGs^11^, including *HOXA1*, *HOXA3*, *HOXA5*, *HOXB4* and *HOXD*, exhibited primed promoters in SC OLGs but not in cortical OLGs, where they remain H3K27me3-repressed (Fig. 7a). These genes are expressed in posterior fossa group A ependymomas (PFA-EP) tumors and H3.1/H3.3K27M pontine HGG, but not more anterior H3.3K27M thalamic HGGs (Fig. 7b and Extended Data Fig. 9c,d). We also analyzed other SC patterning genes (*NKX2-1*, *NKX6-1*, *NOTCH1*, *HES5*, *HES6*, *PAX3*, *PAX6*, *PAX7*, *DBX1*, *IRX3*) and cortical patterning genes (*IRX3*, *LX1*, *DLX2*, *OTX1*, *ZIC1*, *ZIC4*) but found that most retained strong H3K27me3, minimal H3K27ac and ATAC signal across promoters in both regions (Extended Data Fig. 9b).

We then overlaid the chromatin architecture and chromatin immunoprecipitation followed by sequencing (ChIP–seq) data from thalamic and pontine H3.3K27M HGG^11^. Strikingly, pontine HGG presented H3K27ac/H3K27me3 patterns and sub-TAD structures closely resembling those in human SC OLG and hOPCs (Fig. 6c and Extended Data Fig. 9c,d). Interestingly, aggregate loop analysis around the HOX-associated interactions in hOPCs revealed stronger signals in pontine tumors, but could also be observed in the thalamic and posterior fossa tumors (Fig. 7c). Insulation profiles across HOX loci were comparable between hOPCs and all three tumor types and distinct from B cells (Fig. 7d), although the H3K27me3/H3K27ac distributions varied across tumors (Extended Data Fig. 9c,d). Collectively, these findings suggest that the primed state of these genes in the nondiseased context in OLG in the posterior CNS may drive their expression upon PRC2 disruption in distinct brain tumors, being in line with the regional identity of the cell of origin in HGGs being a key determinant in their arisal^11^.

## Discussion

In this study, we profiled single-cell chromatin accessibility and histone modifications across adult human motor cortex, CB and CSC, generating large-scale snATAC–seq and nanoCUT&Tag datasets and high-resolution Micro-C maps in human OPCs. These integrated epigenomic resources enabled the identification of a previously uncharacterized SOX10 enhancer active in the OLG lineage and provided a framework to investigate lineage-specific and region-specific regulatory states.

We identified elevated chromatin accessibility at *HOXA* and *HOXD* clusters in SC OLGs, despite low transcript levels. Accessibility and H3K27ac were concentrated at the 3′ ends, whereas a sharp transition to H3K27me3-enriched chromatin defined the 5′ regions. This pattern suggests that subsets of HOX genes remain in a primed, developmentally derived state. Priming was evident in OLGs and astrocytes but absent in microglia, consistent with their distinct developmental origins. OLGs and astrocytes arise from neuroectoderm that undergoes HOX-dependent patterning, whereas microglia originate from the yolk sac after this patterning is complete, likely explaining the lack of priming in microglia. Epigenetic priming is commonly associated with pluripotent cells^72^. However, we have recently shown that mouse OPCs can activate immune genes in response to an inflammatory insult and that these immune genes are also maintained in a primed state, potentially to enable rapid activation^73–75^.

HOX genes are well studied in development and remain expressed in various adult lineages^76,77^. In the oligodendrocyte lineage, however, their patterning identity is largely lost postnatally, with Hox transcripts actively downregulated^78^, and expressed at lower levels than in neurons^79^, suggesting different mechanisms of regulation of Hox genes in different neural lineages. In contrast, Hox gene activation is strongly correlated with various cancers^61^. We observed that chromatin architecture around HOX clusters in SC OLGs closely resembled that of H3.3K27M pontine pediatric HGG cells, arising from OPCs. Promoter priming and long-range enhancer contacts at genes activated in pons HGG suggest these loci are poised for activation in posterior OLGs but not in anterior regions such as cortex, where H3K27me3 likely enforces repression. Thalamic HGGs may instead rely on anterior developmental regulators such as *OTX1*, *ZIC1* and *ZIC4* (ref. ^11^). While some pons-expressed HOX genes were primed in SC OPCs, others were not, likely reflecting regional differences and additional effects of the H3K27M mutation that are not seen in the nonmutant SC OPCs^80,81^. Nonetheless, the reduced H3K27me3 levels around HOX genes activated in glioma support the idea that primed chromatin states may facilitate reactivation of a subset of these genes in pontine HGGs. However, other regulatory mechanisms beyond those studied here may also be involved in HOX activation and control in adult OLGs.

Our data also suggest that HOX priming may be deleterious, as aberrant HOX activation contributes to tumorigenesis. Within the active *HOXD* TAD, we observed contact between *HOXD3* and *LINC01116*, whose enhancer can drive astrocyte hyperproliferation when activated, a glioma-like phenotype^68^. We show that this enhancer functionally regulates 3′ *HOXD* genes in hOPCs, with both activation and repression altering expression within the same t-Dom but not the adjacent c-Dom, indicating a stable, domain-restricted regulatory architecture. This suggests that the pathway may become activated only in specific cellular contexts.

While our data outline a mechanism for HOX activation in disease, they do not explain why epigenetic memory is maintained; it could simply reflect retained signal from the putative cells of origin, as suggested earlier^11^. Our findings build upon previous work demonstrating that H3K27me3 is crucial for keeping HOX genes inactive and preserving positional identity^76,82,83^. We previously found that its depletion derepresses HOX genes in neonatal mouse OPCs^74^. The simultaneous accessibility and H3K27me3 we observe likely represent a poised, primed state in SC OPCs, although promoter-proximal paused RNA polymerase II may also contribute to maintaining accessibility at these developmental loci^84^. HOX genes are re-expressed in regenerative contexts, including limb injury, where they are strongly upregulated in regenerative stem cells^84^. Regeneration is closely linked to high proliferative capacity, and OPCs are both progenitor-like and highly proliferative, particularly after demyelinating injury^85^. This may underlie the pronounced HOX priming we observe in SC OPCs, which are more proliferative than those in the brain^86^. Consistent with this, the predicted HOX TF activity from our TF network is higher in OPCs than in MOLs, suggesting that priming may support rapid gene activation during regeneration or remyelination and diminishes with differentiation.

In conclusion, this study provides a comprehensive view of chromatin regulation in adult human neural cells and shows that developmental epigenetic states can persist in adult glia. These latent programs may support regenerative competence but may also predispose cells to aberrant activation in tumorigenic contexts.

### Limitations of the study

Our multiome data provide insights into the single-cell histone PTM landscapes of human neural cells in the adult CNS, particularly in the motor cortex and the CSC, revealing region-specific regulation of developmental genes. We identify TFs that act as new putative regulators of specific CNS lineages, which nevertheless will need to be validated with functional experiments in future studies. While our CRISPRa assays support *SOX10* enhancer activity, full functional characterization may require a targeted deletion of the enhancer. Our findings highlight that mapping of histone PTMs in different regions is relevant, and it would be of interest to investigate differences between further posterior CNS regions, such as thoracic and lumbar SC, and other anterior brain regions, such as the thalamus and hippocampus, among others. As our findings reveal developmental epigenetic memory relevant for HGG, probing other CNS regions might reveal additional disease susceptibilities.

Our nanoCUT&Tag dataset exhibits increased sparsity compared to our previously published mouse dataset, although we attribute this disparity to the inherent challenges of working with frozen archival tissue, as opposed to fresh mouse tissue. While canonical bivalency refers to the co-occurrence of H3K27me3 and H3K4me3, we find H3K27ac and H3K27me3 at the same genomic locus. However, higher-resolution methods would be needed to ascertain whether the modifications were found on separate H3 tails of the same nucleosome.

Our chromatin architecture data suggest that the *HOXA* and *HOXD* clusters may also be divided into two separate domains, which are reflective of the active chromatin architecture seen in development when these genes are being expressed. Interestingly, the strong recapitulation of the spatiotemporal context of HOX gene expression in our chromatin data suggests that there might be a role for epigenetic memory, wherein the cells retain some memory of where they came from. Nevertheless, our Micro-C data have high resolution but are still acquired at the bulk level. Moreover, the human iPSC-derived human OPCs are patterned for forebrain identity. Inducing a more posterior identity in iPSC-derived human OPCs or probing the chromatin architecture of human OLGs across different regions of the adult CNS could further elucidate the extent to which the mechanisms described here are broad.

## Methods

### Human tissue collection and processing strategy

Adult postmortem fresh-frozen tissue was obtained from the MRC Sudden Death Brain Bank in Edinburgh with full ethical approval (16/ES/0084) and consent. Work in Sweden was performed under the ethical permit 2016/589-31, with amendment 2019-01503, granted by the Swedish Ethical Review Authority (EPM). Tissue was collected from 20 different donors (*n* = 10 male, *n* = 10 female) within the ages of 34–74 years old (Supplementary Table 1). No statistical method was used to predetermine sample size. Data collection and analysis were not performed blind to the experimental conditions. Each donor donated fresh-frozen WM from the following three tissue regions: primary motor cortex (BA4), arbor vitae cerebelli and fasciculi cuneatus and gracilis from the CSC. Tissue was processed semirandomly, ensuring that each batch of experiments included both sexes and all three tissue regions. Completed libraries were again randomly multiplexed during sequencing to minimize batch effects.

### Tissue dissociation and nuclei isolation

A total of 50–100 mg of frozen tissue were placed in a 1.5 ml tube and chilled in a mortar using liquid nitrogen. A chilled pestle was used to crush the tissue, followed by resuspension in 500 µl of nuclei permeabilization buffer (5% BSA, 0.2% IGEPAL, 1 mM DTT, 1× ethylenediaminetetraacetic acid (EDTA)-free protease inhibitor in PBS). Resuspended tissue was kept on ice for 15 min, with gentle pipetting every 5 min. The homogenized suspension was filtered through a 30 µm filter, followed by a 10 µm filter. An equal volume of 50% iodixanol solution was added and mixed thoroughly. Five-hundred microliter of 29% iodixanol were gently underlaid using a syringe and needle, forming two phases. Samples were centrifuged at 13,500*g* for 20 min at 4 °C. Supernatant was removed and the nuclear pellet was resuspended in wash buffer (2% BSA in PBS). Samples were spun at 1,000*g* for 5 min at 4 °C. Supernatant was discarded and the pellet was resuspended in 30 µl 1× diluted nuclei buffer (DNB; snATAC–seq; 10x Genomics) or 30 µl 1× antibody buffer (nanoCUT&Tag; recipe shown in corresponding section) or 30 µl 1× DNB with 1 U µl^−1^ RNase inhibitor (multiome; 10x Genomics).

### Nano-Tn5 purification and loading

Nanobody-Tn5 fusion proteins were purified as described earlier^29^. Purified enzyme was loaded using barcoded oligonucleotides. First, an equimolar mixture of 100 µM Tn5_P5_MeA_BcdX_0N, Tn5_P5_MeA_BcdX_1N, Tn5_P5_MeA_BcdX_2N and Tn5_P5_MeA_BcdX_3N was mixed with an equimolar amount of 100 µM Tn5_Rev oligonucleotide. The oligonucleotide mixture was denatured by incubating at 95 °C for 5 min in a thermocycler and allowed to anneal slowly by ramping down the temperature by 0.1 °C s^−1^. Furthermore, 8 µl annealed oligonucleotide, 42 µl glycerol, 44.1 µl 2× dialysis buffer (100 mM HEPES-KOH, pH 7.2, 200 mM NaCl, 0.2 mM EDTA, 2 mM DTT (freshly added), 0.2% Triton X-100, 20% glycerol) and 5.9 µl antimouse nano-Tn5 (5 mg ml^−1^, 67.7 µM) were combined to obtain a final volume of 100 µl. Alternatively, 8 μl annealed oligonucleotides, 42 μl glycerol, 45.7 μl 2× dialysis buffer and 4.3 µl antirabbit nano-Tn5 (6.8 mg ml^−1^, 93 µM) were mixed to the same final volume.

### snATAC–seq library preparation and sequencing

Dissociated nuclei were counted and incubated at 37 °C for 60 min in the tagmentation mix. Tagmented nuclei were loaded onto the Chromium Chip H (10x Genomics) according to the manufacturer’s instructions. The Chromium Single Cell ATAC Library and Gel Bead Kit (v1.1; 10x Genomics) was used to generate single-nuclei libraries. All libraries were sequenced on the Illumina NovaSeq 6000 with either the S Prime, S1 or S2 flow cell and a 50-8-16-49 read setup.

### nanoCUT&Tag

Multinano-CUT&Tag libraries were prepared as described earlier. Briefly, dissociated nuclei in antibody buffer (20 mM HEPES, pH 7.5, 150 mM NaCl, 0.05 mM spermidine, 1× protease inhibitor, 0.05% digitonin, 0.01% IGEPAL, 2% BSA, 2 mM EDTA in dH_2_O) were counted and 80,000–120,000 nuclei transferred to 0.5-ml microfuge Eppendorf tubes. Nuclei were topped up to 96 µl with antibody buffer. Mouse H3K27me3 antibody (1:100; Abcam, ab6002), rabbit H3K27ac antibody (1:100; Abcam, ab177178), barcoded antirabbit nano-Tn5 (1:100) and barcoded antimouse nano-Tn5 (1:100) were added to the nuclear suspension (final volume of 100 µl). Samples were then incubated overnight at 4 °C on a rotator. After overnight incubation, cells were centrifuged at 600*g* for 3 min and washed twice with Dig-300 buffer (20 mM HEPES, pH 7.5, 300 mM NaCl, 0.5 mM spermidine, 1× protease inhibitor, 0.05% digitonin, 0.01% IGEPAL, 2% BSA in dH_2_O). After the second wash, nuclei were resuspended in 100-µl tagmentation buffer (20 mM HEPES, pH 7.5, 300 mM NaCl, 0.5 mM spermidine, 1× protease inhibitor, 10 mM MgCl_2_, 0.05% digitonin, 0.01% IGEPAL, 2% BSA in dH_2_O) and incubated at 37 °C for 60 min. Tagmentation was stopped by adding 100-µl STOP buffer (12.5 mM EDTA in 1× DNB (10x Genomics) supplemented with 2% BSA). Nuclei were centrifuged at 600*g* for 3 min and washed twice with 1× DNB/BSA to remove traces of EDTA. After the second wash, 185 µl of supernatant was removed and nuclei were resuspended in the remaining 15 µl. A total of 2 µl were used for counting (1:5 diluted in trypan blue).

### nanoCUT&Tag library preparation and sequencing

Single-cell indexing was performed according to Chromium Next GEM Single Cell ATAC Library and Gel Bead Kit (v1.1; 10x Genomics) instructions. Nuclei of 8 µl were added to 7 µl ATAC buffer B (10x Genomics) and loaded onto the Chromium Chip H. GEM incubation and post-GEM incubation clean-up were performed according to Chromium Next GEM Single Cell ATAC Reagent Kits (v1.1) instructions (step 2.0–3.2). Of the 40 µl of eluted sample, 2 µl was used to measure the concentration using the Qubit dsDNA HS Assay kit. The remaining sample was used for P7 tagmentation by mixing with a tagmentation reaction containing 2× TD buffer (20 mM Tris, pH 7.5, 20% dimethylformamide, 10 mM MgCl_2_), 1 µl of 10 ng µl^−1^ MeB-loaded standard Tn5 and dH_2_O to a final volume of 100 µl, followed by incubation at 37 °C for 30 min in a thermocycler. After tagmentation, samples were purified using DNA Clean and Concentrator-5 (Zymo) according to the manufacturer’s instructions and eluted in 40 µl Zymo elution buffer. Purified DNA was used as input for the Sample Index PCR in the Chromium Next GEM Single Cell ATAC Reagent Kits (v1.1; step 4.1) and samples were amplified for 11–15 cycles. After the Sample Index Double-Sided Size Selection was performed according to the manufacturer’s instructions. Library quality was checked on the Agilent bioanalyzer and sequenced on the Illumina NovaSeq 6000 S Prime flow cell (100c kit) with a custom read1 (R1_seq, 5′-GCGATCGAGGACGGCAGATGTGTATAAGAGACAG-3′) primer, custom index2 (I2_seq, 5′-CTGTCTCTTATACACATCTGCCGTCCTCGATCGC-3′) primer and a 36-8-48-36 read setup.

### Multiome library prep

Tissue dissociation and nuclei extraction were performed as described above. A total of 10,000 nuclei were counted and used for bulk tagmentation followed by loading on the Chromium Next GEM Single Cell Chip J. Single-cell indexing and library preparation were performed using the Chromium Next GEM Single Cell Multiome ATAC + Gene Expression kit, according to the manufacturer’s instructions. Libraries were sequenced on the Illumina NovaSeq 6000 S Prime flow cell (100c kit), with a 50-8-24-49 read setup.

### iPS-derived hOPC cell cultures

hOPCs were derived from the human iPS line C27 (ref. ^87^) in Steven Goldman’s lab, with the protocol described in ref. ^51^. Work in Sweden was performed under the ethical permit 2020-00398, with amendment 2023-04598-02, granted by the Swedish EPM. Corning six-well cell culture plates were precoated with poly-L-ornithine (PLO; Sigma-Aldrich, P4957-50ML) and incubated for 1 h at 37 °C. PLO was removed and wells were rinsed thrice using sterile 1× DPBS^−/−^ (Thermo Fisher Scientific, 14190144) followed by overnight incubation with 5 µg ml^−1^ laminin (Corning, 354232) in HBSS^+/+^ (Thermo Fisher Scientific, 24020117). After removing the laminin, 1 million iPS-derived hOPC cells (C27 line) were directly seeded into the plate and expanded for 3 weeks before splitting. Cells were cultured in proliferation media (DMEM/F12 (Invitrogen, 11330-057) containing 1× B27 (Invitrogen, 12587-010), 1× N1 (Sigma-Aldrich, N6530), 1× NEAA (Invitrogen, 11140-050), 60 ng ml^−1^ T3 (Sigma-Aldrich, T5516-1MG), 1 µM dcAMP (Sigma-Aldrich, D0260), 100 ng ml^−1^ biotin (Sigma-Aldrich, B4639), 10 ng ml^−1^ PDGF-AA (R&D, 221-AA-50), 10 ng ml^−1^ IGF-1 (R&D, 291-G1-050) and 10 ng ml^−1^ NT3 (R&D, 267-N3-025)), which was refreshed every 2 days.

### ATAC–seq in hOPCs

ATAC–seq was performed as described previously^7^. A total of 60,000 cultured hOPCs were collected, washed with 1× PBS, and incubated in lysis buffer (0.1% IGEPAL, 10 mM Tris–HCl, pH 7.4, 10 mM NaCl, 3 mM MgCl_2_) on ice for 5 min. Lysed cells were centrifuged at 500*g* for 20 min at 4 °C. The nuclei pellet was resuspended in tagmentation mix (2× TD buffer, Tn5 enzyme, in dH_2_O) and incubated at 37 °C for 30 min. Tagmented DNA was purified using the Qiagen minElute Purification kit and PAGE purified to remove adaptor dimers. Libraries were sequenced on an Illumina NovaSeq 6000 with a 50-8-8-50 read setup.

### B cell collection

Peripheral mononuclear cells were freshly isolated by Ficoll (GE Healthcare, 17-1440-03) gradient centrifugation from buffy coats obtained through Karolinska University Hospital of three healthy female donors (age = 28, 29 and 39 years). Study procedures were conducted under ethical permit 2009/2107-31-2 approved by the Swedish EPM. B cells were then enriched by negative selection using the EasySep Human B Cell Enrichment Kit II (STEMCELL Technologies, 17963) without CD43 depletion, according to the manufacturer’s instructions. B cells were then stained for 30 min on ice with anti-CD3 (clone SK7; 560176), anti-CD14 (clone MφP9; 560180), anti-CD16 APC-Cy7 (clone 3G8; 560195), anti-CD19 APC (clone HIB19; 561742), anti-IgG BV510 (clone G18-145; BD Biosciences, 563247), anti-CD27 PE-Cy5.5 (clone 0323; Novus Biologicals, NBP1-43426) or BV711 (clone O323; 302833), anti-IgD pacific blue (clone IA6-2; 348224), anti-IgM BV570 (clone MMH-88; 314517) and Zombie NIR fixable viability dye (BioLegend, 423106). Cells were then washed in PBS (Sigma-Aldrich, D8537) and filtered. Between 10^6^ and 2 × 10^6^ CD27^+^CD19^+^ live B cells were sorted using a SH800 Cell Sorter (Sony) into an RPMI medium (R8758) with 10% heat-inactivated fetal bovine serum (F7524), 100 U ml^−1^ penicillin and 100 μg ml^−1^ streptomycin (Sigma-Aldrich, P4458). Cells were washed in PBS, centrifuged and stored at −80 °C as dry pellet before proceeding with Micro-C.

### Processing of human prefrontal cortex biopsy for scRNA-seq

The scRNA-seq prefrontal cortex sample was selected from a cohort of patients with hydrocephalus, a 76-year-old female, planning to undergo cerebrospinal fluid (CSF) diversion surgery either with a ventriculoperitoneal shunt placement or ventriculocisternostomy and without diagnosed CNS malignancy, hematoma, infection or inflammation. Patients received oral and written information and were required to sign an informed consent. The biopsy procedure was performed by a standard operative procedure under general anesthesia. The placement of burr hole on the frontal lobe followed by an opening of dura and cortex was performed in a routine fashion for ventriculoperitoneal shunt or ventriculocisternostomy surgery. The biopsy was sharply dissected with knife through the leptomeninges, forming a tissue block consisting of cortex and subcortex, and placed in a sterile tube containing CSF. Biopsies were immediately placed on ice and transported to the nearby laboratory. After biopsy was taken, hemostasis was achieved using diathermy and the CSF diversion surgery continued. The wound was closed routinely. No surgical insults were detected in the study population. Patients and relatives received oral and written information about the study before inclusion and provided signed informed consent on hospital admission. Patients who did not wish to participate or were unable to understand the information or provide signed informed consent were excluded. Patient data and samples were anonymized for the research group. The study was approved by the Stockholm Region’s ethical committee (2016/1062-31/2 and 2018/843-32). Fresh tissue sample processing was performed within the hours before surgery. The tissue was dissociated, followed by red cell and debris removal, and cell counting for sequencing (scRNA-seq; 10x Genomics). After dissociation, only non-neuronal cells were retrieved.

FASTQ files of prefrontal cortex scRNA-seq were processed using Cellranger count (v6.12) and aligned to the GRCh38 genome assembly. Gene count matrix was processed with Seurat (v4). For QC filtering and downstream dimension reduction and clustering, cells with filters nFeature_RNA > 200 & nFeature_RNA < 10,000 & percent_mito ≤ 5 were retained, which led to a final dataset of 2,798 cells. Cells were annotated using canonical markers and compared to the full scATAC–seq dataset and multiome datasets from this study, using the gene activities from the scATAC–seq as a proxy of the RNA expression. The final dataset included different non-neuronal cell types, 186 astrocytes (ASTRO), 714 microglia (MIGL), 1,826 oligodendrocytes (OL) and 72 OPCs. Integration with scATAC–seq cortex data was performed with Seurat (v4) FindTransferAnchors on the intersecting variable features (from the top 3,000 most variable features). Anchors were identified between the scRNA-seq prefrontal cortex genes and the gene activities from the corresponding genes on the scATAC–seq dataset. Predicted labels were transferred using Labeltransfer with dims = 1:20 and weight.reduction = ‘cca’.

### Micro-C

Micro-C was performed by the National Genomics Infrastructure, Stockholm, Sweden, using the commercially available Micro-C kit (Dovetail Genomics, 21006), with 100,000–200,000 iPS-derived hOPCs as input. Briefly, cells were crosslinked and enzymatically digested using MNase to allow for nucleosome-resolution fragmentation. Free ends were ligated with biotin-containing adaptors. Ligated fragments were reverse-crosslinked and amplified to introduce sequencing handles. Libraries were generated in three separate biological replicates and were sequenced as a pilot on the Illumina NovaSeq 6000 S Prime flow cell with a 2 × 150 bp (300c) kit with a 151-19-10-151 read setup. After QC of the pilot and checking for library complexity (with the ‘preseq’ tool), two of three hOPC replicates and all three B cell replicates were resequenced on a large S4 flow cell to a depth of 6 billion reads.

### gRNA cloning strategy

Four guides per locus were cloned by annealing 1 µl of each complementary oligo (Supplementary Table 5) to a final volume of 25 µl annealing buffer (combine 500 µl 1 M Tris–HCl, pH 8.0, with 500 µl 5 M NaCl to final volume of 50 ml with ddH_2_O) and incubated at 95 °C for 3 min, cooled to 22 °C at 0.1 °C s^−1^ and diluted in 75 µl ddH_2_O to yield 1 µM. Lentiviral U6–GG–acceptor plasmid was prelinearized using BsmBI (20 U µl^−1^; NEB, R0580) and BsiWI-HF (20 U µl^−1^; NEB, R3553) and isolated on gel and aliquoted at 30 ng µl^−1^. T4 ligation was carried out mixing 1 µl of 1 µM annealed oligos, 30 ng linearized GG–acceptor plasmid, 0.2 µl BsmBI (20 U µl^−1^), 0.2 µl BsiWI-HF (20 U µl^−1^), 0.5 µl T4 DNA ligase (40 U µl^−1^; NEB, M0202), 1 µl 10× T4 DNA ligase buffer and 6.1 µl ddH_2_O and incubating for 10 min at 20 °C, followed by 5 min at 37 °C and 5 min at 80 °C. One microliter of the ligation reaction was added to 10 µl of competent cells and incubated on ice for 10 min. Cells were heat-shocked at 42 °C for 30 s, returned to ice for 5 min, and then supplemented with 100 µl SOC medium. The cells were incubated at 37 °C for 60 min with shaking at 250 rpm and plated on agar plates containing ampicillin for overnight selection. Colonies were isolated and expanded in liquid LB medium prior to plasmid isolation using the ZymoPURE Plasmid Miniprep Kit (Zymo Research, D4210) according to the manufacturer’s instructions. Purified plasmids were verified by Sanger sequencing using the primer 5′-CGATACAAGGCTGTTAGAGAG-3′.

### Lentiviral packaging

dCas9–KRAB–BFP, dCas9–p300–BFP and U6–GG–acceptor lentiviral plasmids were a gift from CRISPR Functional Genomics, Karolinska Institute. Lentivirus was packaged in HEK293 cells cultured in DMEM (Gibco, 31966021) supplemented with 10% FBS (Gibco, 10500064) on precoated poly-l-lysine (Sigma-Aldrich, P4707) culture ware seeded to be 70–80% confluent after >24 h. A total of 16 µg of transfer vector, pCMV-VSV-G, pRSV-REV and pCgpV (Takara, 631278) were mixed at a ratio of 3:1:1:1 with 32 µl lipofectamine-2000 (Thermo Fisher Scientific, 11668030) and Opti-MEM (Gibco, 31985070) according to the manufacture’s protocol and added to the cells. Viral particles were collected from the media after 48 h, centrifuged at 500*g* for 10 min and filtered through 0.45 µm before incubating with Lenti-X concentrator (Takara, 631231) at a ratio of 3:1 and incubated for >30 min at 4 °C, before centrifugation at 1,500*g* for 45 min at 4 °C and resuspended in DMEM at a 20× or 100× concentration.

### Lentiviral transduction of hOPCs

Concentrated lentivirus was added for <24 h after seeding to the media at a ratio of 1:4:1:3, and growth factors and supplements were adjusted accordingly. dCas9–BFP and gRNA-mCherry transduced hOPC at a rate of 25–20% and >90%, respectively. After 24h, the media was replaced, and cells were kept for 3 days before collecting using miRNeasy Micro Kit (Qiagen, 217084) according to the manufacturer’s protocol. cDNA library was prepared using High-Capacity cDNA Reverse Transcription Kit (Thermo Fisher Scientific, 4368814). RT-qPCR was performed on a StepOnePlus System (Applied Biosystems) in triplicate and with reverse transcriptase-negative reactions to control for genomic DNA. Fast SYBR Green Master Mix (Applied Biosystems, 4385616) was used according to the manufacturer’s protocol, each PCR reaction had a final volume of 10 µl and 2 µl of diluted cDNA and reverse transcriptase, respectively, and incubated at 20 s at 95 °C, 40× cycles of 3 s of 95 °C and 30 s of 60 °C, followed by 15 s at 95 °C, 60 s at 60 °C and 15 s at 95 °C. Melting curve was obtained for each PCR product after each run, to control for primer dimers and gene-specific peaks. Expression levels were calculated by dividing the quantity by the geometric mean of the housekeeping genes.

### RNA extraction, cDNA synthesis and qRT–PCR

Cells were collected with QIAzol (Qiagen) and stored at −80 °C until further processing. RNA was extracted with the miRNeasy mini kit (Qiagen) according to the manufacturer’s instructions. Contaminating DNA was degraded by the treatment of the samples with RNase-free DNase (Qiagen) in column. Thus, 350 ng RNA was used to synthesize cDNA with the High-Capacity cDNA Reverse Transcription Kit (Applied Biosystems), including RNase inhibitor (Applied Biosystems), with annealing for 10 min at 25 °C and extending for 2 h at 37 °C and inactivation for 5 min at 85 °C. The cDNA was diluted 1:5 in H_2_O and 2.5 μl was used in the qRT–PCR reactions with 2 μl of Fast SYBR Green Master Mix (Applied Biosystems) and 5 pmol of each primer in a final volume of 10 μl. The reactions were run on a StepOnePlus System (Applied Biosystems) in duplicate and with reverse transcriptase-negative reactions to control for genomic DNA. The running conditions were 20 s at 95 °C, followed by 40 cycles of 3 s of 95 °C and 30 s of 60 °C, then 15 s at 95 °C, 1 min at 60 °C and 15 s at 95 °C. Melt curves were generated to control primer dimers and gene-specific peaks. Relative standard curves for each gene were generated to obtain relative expression values. ‘GAPDH’ and ‘b-Act’ were run as housekeeping genes. Expression levels were then calculated by dividing the relative expression value by the geometric mean of the housekeeping genes. Samples were normalized per experiment. Data distribution was assumed to be normal, but this was not formally tested.

### snATAC–seq data preprocessing and QC

FASTQ files generated from sequencing were processed using ‘cellranger-atac count’ with the default parameters. Samples were aggregated using the ‘cellranger-atac aggr’ with default parameters, but with the normalization omitted using the flag ‘—normalize=none’.

Thus, 2-kb count matrices were built using a custom script ‘build_large_mtx.py’ that is a modified version of episcanpy’s^88,89^ build_atac_mtx.py script and allows for reading in files in batches. TSSe scores were generated using the ArchR^90^ package and only cells with TSSe >7 and number of unique fragments of >3,000 were retained.

### snATAC–seq peak calling

The Fragments file (fragments.tsv.gz) was split according to the cell-type annotation and peaks were called using the ‘callpeak’ function from MACS2 (ref. ^91^) with the following parameters: ‘-f BED -g hs -q 0.05 –shift -100 –extsize 200 –nomodel –call-summits –keep-dup = 1’. Peak annotation was performed using the HOMER^92^ annotatePeaks.pl function and a custom GTF file with miRNA and snoRNA removed.

### snATAC–seq downstream analysis

After cell filtering, the top 100,000 features were retained and normalization was performed using term frequency-inverse document frequency, followed by singular vector decomposition. A nearest-neighbor graph was built in the lower-dimensional space, followed by Leiden clustering. The highly variable features from each cluster were retained and used to repeat the term frequency-inverse document frequency, singular vector decomposition, graph building and clustering for a total of three iterations. Batch correction was performed using Harmony^93^. Differentially accessible regions were identified using both the ‘rank_features’ function in episcanpy (v0.3.2) with Benjamini–Hochberg correction for multiple testing as well as the diffxpy (https://github.com/theislab/diffxpy/) package with sex, age and tissue added as covariates.

### Gene activity matrix and cell-type annotation

A gene activity matrix was built using a 5 kb promoter region flanking the TSS of genes. The count matrix was smoothened using MAGIC^94^ with default parameters to improve the signal. The top 50 distinct differentially accessible genes for all cell types found in our published snRNA-seq dataset were used as cell-type metagenes and aggregate gene activity was calculated for all genes within each cell-type metagene, generating a metagene score for each cell. Metagene scores for the different cell types and individual marker genes were used to assign the clusters to broad cell types. We could not identify SC-derived neurons, although it is known that these neurons are particularly sensitive and susceptible to hypoxia, possibly leading to difficulty in isolating them. An overwhelming proportion of all cerebellar cells in the dataset was composed of the CBEX cells (also known as cerebellar granular cells). These cells are tiny and densely packed within the granular layer of the CB, close to the gray matter–WM border. We suspect that the skewed distribution may have arisen from imprecise dissection during the collection of WM from the tissue.

### Integration with snRNA-seq data

The annotation of the cell types based on transcriptome data from external datasets was performed as discussed in refs. ^5,37^. The expression matrix mentioned in the ref. ^37^ was downloaded from Gene Expression Omnibus repository with the accession GSE118257, and then converted to Seurat^95^ (v4.3.0.1) object. The dataset mentioned in ref. ^5^ was downloaded from https://cellxgene.cziscience.com/collections/9d63fcf1-5ca0-4006-8d8f-872f3327dbe9 as a Seurat object including all the cell types. scATAC h5ad file was converted to Seurat using SeuratDisk R library with the Convert function with assay = ‘peaks’ to h5Seurat format. Gene activities were calculated based on the chromatin accessibility signal using ENSEMBL gene annotations (EnsDb.Hsapiens.v86). For each annotated gene, the region of the promoter (500 bp upstream of the annotated TSS) and the gene body were considered (promoter + genebody). Gene activities were calculated using FeatureMatrix function from Signac^96^ (v1.10.0) with the promoter + genebody in GRanges format, from GenomicRanges^97^ library, and the cellranger-atac 2.0 fragments.tsv for all the samples. Then, they were added to the Seurat/Signac object as a new assay. Label transfer was performed using Seurat. The FindIntegrationAnchors function was applied to find anchors between scRNA reference dataset and the query scATAC gene activities. The FindTransferAnchors function was used to find transfer anchors from each reference to the query, and then the resulting anchors were used to perform label transfer with the TransferData function, using canonical correlation analysis with 20 dimensions.

### Trimodal genomic clustering

A total of 10-kb genomic bins spanning the hg38 genome were used as input peaks to the deepTools^98^ compute-matrix function. ATAC, H3K27ac and H3K27me3 bigwig files from each population were used to generate a matrix of normalized signal in each genomic bin. The mean signal across each bin across each modality and each cell type was used to hierarchically cluster the bins (rows) and celltype + modality (columns). Pearson correlation was used to identify the correlation among each column (identifying similar and dissimilar celltype + modalities based on whole-genome patterns).

### Co-accessibility analysis

Co-accessible regions were identified using Cicero^99^. Cell-type-specific pseudobulk BAM files and single-cell count matrices were provided as input to identify pairs of genomic bins with increased co-accessibility across different cells of a population. Co-accessibility score cutoffs of 0.25 and 0.5 were used to identify significant interactions and high-confidence interactions, respectively.

### TF regulatory network

The Core Regulatory Circuit package^100^ was used to identify the core TF network. Briefly, H3K27ac BAM and bigwig files and Super Enhancers (using ROSE algorithm) for each cell population were used as input to FIMO. FIMO then evaluates TF motif enrichment through a *P*-value cutoff based on a log-likelihood ratio test. For the OPC and CXINH populations, the ATAC–seq data (BAM, bigwig, Super Enhancers) were used instead due to the sparsity of the H3K27ac data^101^. A network was built by inferring the number of interacting TF motifs in the proximal super enhancer of a TF. The TF strength was assigned based on the difference between the number of outbound edges (regulated TFs) and inbound edges (targeting TFs). A higher score suggests that a TF has a stronger regulatory influence over other TFs.

### TF footprinting analysis

Footprinting analysis was performed using the Regulatory Genomics Toolbox^102^. Briefly, peaks were called for the different cell types, and the HMM-based Identification of TF footprints (HINT) framework was applied to identify active TF-binding sites using default parameters.

### nanoCUT&Tag signal enrichment

The *k*-means algorithm (*k* = 10) implemented in deepTools (v3.5.1) was used to cluster all genes (1-kb-padded TSS) based on the H3K27ac and H3K27me3 signals for each cell type. We then queried the genes identified in the clusters that displayed high H3K27ac and low H3K27me3 and used gget enrichr^103^, predicting the enriched cell type based on the genes (database = ‘celltypes’).

### Micro-C balancing and transformation

Raw contact matrices were normalized and balanced using iterative correction and eigen decomposition as implemented in the ‘cooler’^104^ package. The ‘hicTransform’ package was used to generate observed/expected counts with the ‘–method obs_exp’ flag.

### Compartment analysis and aggregate pileup analysis

Micro-C compartments were identified by computing the principal component analysis eigenvectors of the hic matrix using the ‘hicPCA’ command from the hicExplorer package^105–107^. Aggregate pileup was performed using the ‘coolpuppy’ package^108^. Briefly, signal aggregation was done in all hic matrices (hOPCs, B cells, PFA-EP, pontine HGG, thalamic HGG) at genomic coordinates corresponding to the identified loops.

### Insulation and boundary strength analysis

The ‘cooltools’^109^ Python API was used to process the contact matrices and identify the insulation scores within the normalized contact frequency data. Briefly, a diamond-shaped window is used to slide along the genome, with one of the corners on the main diagonal of the contact matrix, and contacts within the window at each position are summed up. Windows with low sums are marked as putative boundaries and as insulating regions immediately upstream and downstream. Boundaries were identified in the 10-kb contact matrix with a sliding window size of 100 kb.

### Loop calling and virtual 4C analysis

Virtual 4C identifies loci that exhibit increased contact frequency with a reference locus of interest (viewpoint analysis) and was performed using the ‘hicPlotViewpoint’ function in the HiCExplorer^106^ package. Loops were called on the 5-kb contact matrix using the ‘mustache’^110^ package and looking within a maximum distance of 100 Mb.

### Additional computational analysis

Description of the following computational analysis is given in Supplementary Methods: motif analysis, integration of multiome ATAC with snATAC–seq data, nanoCUT&Tag data preprocessing and cell calling, nanoCUT&Tag peak calling and bigwig track generation, integration of H3K27ac nanoCUT&Tag with snATAC–seq data, regulatory domain border identification, micro-C data preprocessing and Hi–C to Cool matrix conversion.

### Reporting summary

Further information on research design is available in the Nature Portfolio Reporting Summary linked to this article.

## Online content

Any methods, additional references, Nature Portfolio reporting summaries, source data, extended data, supplementary information, acknowledgements, peer review information; details of author contributions and competing interests; and statements of data and code availability are available at 10.1038/s41593-026-02208-0.

## Supplementary information

Supplementary Information Supplementary Methods.

Reporting Summary

Supplementary Tables Supplementary Table 1: Human tissue metadata. Supplementary Table 2: Co-accessible loci (Cicero) for all cell types. Supplementary Table 3: TF networks for all cell types. Supplementary Table 4: Differentially accessible regions in glia across sex, age and tissue. Supplementary Table 5: gRNA sequences and qPCR primers. Supplementary Table 6: Plasmid sequences for lentiviral constructs.

## Source data

Source Data Figs. 2 and 6 and Extended Data Fig. 6 Statistical source data for Figs. 2 and 6 and Extended Data Fig. 6.

## Data Availability

Raw Human data have been deposited in the European Genome-Phenome Archive (EGA) under EGA accession EGAD50000000410 for the scATAC–seq, nanoCT-seq, SC multiome and hOPCs Micro-C data (EGAD50000001542) for the scRNA-seq from the human prefrontal cortex biopsy and EGAD50000001535 for B cells Micro-C data. Browsable UMAPS and tracks are available at UCSC Cell Browser and UCSC Genome Browser^34^ (https://cns-nanocuttag-atac.cells.ucsc.edu) and https://ki.se/en/mbb/oligointernode. Source data are provided with this paper.

## Code availability

All code is available at https://github.com/mkabbe/snATACnanoCT_AdultHumanCNS.
