# Supplementary material for: Single-nucleus epigenomic profiling of the adult human central nervous system unveils epigenetic memory of developmental programs
Source: Nat Neurosci. 2026 Mar 19;29(4):992–1006. doi: 10.1038/s41593-026-02208-0 (PMC13061643; doi:10.1038/s41593-026-02208-0)
Supplement: Supplementary file 1 — Supplementary Methods. [file 41593_2026_2208_MOESM1_ESM.pdf]

nature neuroscience

Supplementary information

https://doi.org/10.1038/s41593-026-02208-0

Single-nucleus epigenomic profiling of the adult human central nervous system unveils epigenetic memory of developmental programs

In the format provided by the authors and unedited

Single-nucleus epigenomic profiling of the adult human central nervous system unveils epigenetic memory of developmental programs

Mukund Kabbe[1,15], Eneritz Agirre[1], Karl E. Carlström[1], Özge Dumral[1], Yuk Kit Lor[1], Fabio Baldivia Pohl[1], Nicolas Ruffin[2], David van Bruggen[1], Mandy Meijer[1,3], Luise A. Seeker[4,14], Nadine Bestard-Cuche[4], Alex R. Lederer[5], Jilin Zhang[6], Virpi Ahola[6,7], Steven A. Goldman[8,9], Erik Edström[10], Lisa Arvidsson[11,12], Tiago Holm Moreira[10,12], Marek Bartosovic[13], Maja Jagodic[2], Anna Williams[4], Gonçalo Castelo-Branco[1 †]

Author Affiliations

1 – Laboratory of Molecular Neurobiology, Dept. of Medical Biochemistry and Biophysics, Science for Life Laboratory, Karolinska Institutet, Stockholm, Sweden
2 – Dept. of Clinical Neuroscience, Karolinska Institutet, Centre for Molecular Medicine, Karolinska University Hospital, Stockholm, Sweden
3 – Max Delbrück Centre for Molecular Medicine in the Helmholtz Association (MDC Berlin), Berlin, Germany
4 – Centre for Regenerative Medicine, Institute of Regeneration and Repair, University of Edinburgh, Edinburgh, UK
5 – Laboratory of Brain Development and Biological Data Science, School of Life Sciences, Ecole Polytechnique Federale de Lausanne, Lausanne, Switzerland
6 – Ming Wai Lau Centre for Reparative Medicine, Karolinska Institutet, Hong Kong, China
7 – Institute of Biomedicine, University of Eastern Finland, Kuopio, Finland
8 – Centre for Translational Neuromedicine, University of Copenhagen, Copenhagen, Denmark
9 – Centre for Translational Neuromedicine, University of Rochester, Rochester, USA
10 – Department of Clinical Neuroscience, Karolinska Institutet
11 – Department of Women's and Children's Health, Karolinska Institutet
12 – Department of Neurology, Karolinska University Hospital, Stockholm, Sweden
13 – Department of Biochemistry and Biophysics, Stockholm University, Stockholm, Sweden
14 – Department of Bioengineering, Stanford University; Stanford, CA, USA
15 – Current Address: NEXUS Epigenomics AB, Stockholm, Sweden
† – Corresponding author (goncalo.castelo-branco@ki.se)

Supplementary Methods

Motif analysis

For identifying TF motif differences, we used ChromVar, which incorporates per-cell normalization to correct for transposase bias and depth bias, to output a deviation score for each TF motif. Here, we used the version of ChromVar adapted in ArchR allowing scalability to large datasets. We applied the standard ArchR pipeline and calculated the deviation scores using the CIS-BP database as TF binding reference and then exported the motif deviation matrix to CSV format.

Integration of multiOme-ATAC with snATAC-seq data

The ATAC object was subsetted to contain only the same donors in which the multiOme was performed. The ATAC and multiOme-ATAC 2kb objects were concatenated and normalized using TF-IDF, followed by SVD dimensionality reduction. A kNN classifier was used to annotate the cells derived from the multiOme-ATAC according to the closest neighbors found in the annotated ATAC object. Transferred annotations in the multiOme-ATAC object were checked using the RNA expression of marker genes in the multiOme-RNA object.

nanoCUT&Tag data pre-processing and cell calling

Fastq files were split into antibody-specific fastq files using a custom *debarcoding.py* script. Once split, each antibody's fastq file were used as input into 10x Genomics cellranger-atac v2.0 pipeline with the following parameters '*count –id=$(sample) –sample=$(sample) –fastqs=$(sample_fastqs) –reference=cellranger-atac/refdata-cellranger-atac-GRCh38-2020-A-2.0.0.*' The output pseudobulk alignment file from cellranger was used to call peaks with MACS2 algorithm with the following parameters '*callpeaks -g hs –keep-dup=1 –llocal 100000 –min-length 1000 –max-gap 1000*'. We then plotted the fraction of reads in peaks versus the total number of unique reads to custom select barcodes that we consider cells. The 'is_cell_barcode' bit in the singlecell.csv file from cellranger was reset or flipped to represent the called cells.

nanoCUT&Tag peak calling and bigwig track generation

Peaks were called using the MACS2 *callpeak* function with the same parameters described above.

Fragments were split by cell type and used to generate a bam file with the bedtools bedtobam function. Cell type-specific bam files were sorted and indexed prior to bigwig generation. Bigwig files were generated using the following command – "*bamCoverage --normalizeUsing RPKM --binSize 50 --centerReads --smoothLength 250*".

Integration of H3K27ac nanoCUT&Tag with snATAC-seq data

The H3K27ac dataset was first subsetted to include only the cell barcodes shared with the H3K27me3 dataset. A new 2kb-count matrix was constructed for the H3K27ac dataset (query) and only features shared in the filtered ATAC dataset (reference) were retained. Cerebellar cells of the ATAC dataset were removed and the query dataset was integrated to the reference using the Scanpy ingest tool which is based on asymmetric mapping of the query data onto the reference's nearest neighbor graph.

Regulatory domain border identification

Normalized ATAC, H3K27ac and H3K27me3 signal distribution in genomic windows spanning 50kb upstream to 50kb downstream of each HOX cluster was calculated using deeptools compute-matrix. Each window was binned into 10kb bins, and the Kolmogorov-Smirnov test was used to identify pairs of adjacent bins with significantly different signal distribution for each modality separately. The shared border between the identified adjacent bins was considered as a "signal border". Identified signal borders were annotated as weak,

intermediate, or strong depending on if it was identified in only 1, 2 or all 3 modalities. Gaussian smoothening was applied to the signal for visualization in the plots, however all calculations were performed on the raw signal in each window.

Micro-C data pre-processing

Micro-C data was analyzed using the Dovetail Genomics analysis pipeline (github.com/dovetail-genomics/Micro-C/tree/main). The fastq files from the pilot and deep sequencing batch were merged and were aligned to the human genome (GRCh38) using bwa mem with the flags "*-5SP -T0 -t24*". Pairtools was used to parse the aligned reads with the following command "*pairtools parse –min-mapq 40 –walks-policy 5unique –max-inter-align-gap 30 –nproc-in 16 –nproc-out 16*". The count matrix in the .hic format was generated using the juicer package: "*java -Xmx32g -Djava.awt.headless=true -jar juicertools.jar pre –threads 16*". Quality check of reads was performed using the get_qc.py script in the dovetail pipeline.

HiC to Cool matrix conversion

.hic files are multi-resolution files and can be converted using *hic2cool* to generate multi-resolution .mcool files. However, due to the lack of updates in the hic2cool package, a workaround script "convertHic2Cool.py" was used which is an adaptation of the code sourced from (github.com/deeptools/HiCExplorer/issues/821#issuecomment-1316842070) and allows for generation of single resolution *.cool* files. Due to the space inefficiencies of storing multiple .cool files at all resolutions, individual cool files were generated from the parent .hic file for different analyses.
