# Supplementary material for: Single-nucleus epigenomic profiling of the adult human central nervous system unveils epigenetic memory of developmental programs
Source: Nat Neurosci. 2026 Mar 19;29(4):992–1006. doi: 10.1038/s41593-026-02208-0 (PMC13061643; doi:10.1038/s41593-026-02208-0)
Supplement: Supplementary file 2 — Reporting Summary [file 41593_2026_2208_MOESM2_ESM.pdf]

nature portfolio

Corresponding author(s): Gonçalo Castelo-Branco

Last updated by author(s): Dec 4, 2025

Reporting Summary

Nature Portfolio wishes to improve the reproducibility of the work that we publish. This form provides structure for consistency and transparency in reporting. For further information on Nature Portfolio policies, see our Editorial Policies and the Editorial Policy Checklist.

Statistics

For all statistical analyses, confirm that the following items are present in the figure legend, table legend, main text, or Methods section.

n/a	Confirmed	
☐	☒	The exact sample size (*n*) for each experimental group/condition, given as a discrete number and unit of measurement
☐	☒	A statement on whether measurements were taken from distinct samples or whether the same sample was measured repeatedly
☐	☒	The statistical test(s) used AND whether they are one- or two-sided<br>*Only common tests should be described solely by name; describe more complex techniques in the Methods section.*
☐	☒	A description of all covariates tested
☒	☐	A description of any assumptions or corrections, such as tests of normality and adjustment for multiple comparisons
☐	☒	A full description of the statistical parameters including central tendency (e.g. means) or other basic estimates (e.g. regression coefficient) AND variation (e.g. standard deviation) or associated estimates of uncertainty (e.g. confidence intervals)
☐	☒	For null hypothesis testing, the test statistic (e.g. *F*, *t*, *r*) with confidence intervals, effect sizes, degrees of freedom and *P* value noted<br>*Give P values as exact values whenever suitable.*
☒	☐	For Bayesian analysis, information on the choice of priors and Markov chain Monte Carlo settings
☒	☐	For hierarchical and complex designs, identification of the appropriate level for tests and full reporting of outcomes
☐	☒	Estimates of effect sizes (e.g. Cohen's *d*, Pearson's *r*), indicating how they were calculated

Our web collection on statistics for biologists contains articles on many of the points above.

Software and code

Policy information about availability of computer code

Data collection	Data was collected using standard Illumina sequencing with standard software.<br><br>snATAC-seq: raw fastq files were processed using cellranger-atac 1.2.0.;<br>multiOme libraries: raw fastq files were processed using cellranger-arc 2.0.2;<br>nanoCUT&Tag fastq files were first demultiplexed into modality-specific fastq files using the debarcode.py script, with 1 mismatch in the barcode allowed. Demultiplexed fastq files were then. Processed using cellranger-atac 2.1.0.;<br>Micro-C libraries were processed using the dovetail genomics pipeline available at: https://micro-c.readthedocs.io<br><br>Data was analyzed using combination of published tools and custom scripts. All code needed to reproduce the analysis and figures are available at https://github.com/mkabbe/snATACnanoCT_AdultHumanCNS
Data analysis	Data was analyzed using combination of published tools and custom scripts. All code needed to reproduce the analysis is available at https://github.com/mkabbe/mkabbe/snATACnanoCT_AdultHumanCNS

For manuscripts utilizing custom algorithms or software that are central to the research but not yet described in published literature, software must be made available to editors and reviewers. We strongly encourage code deposition in a community repository (e.g. GitHub). See the Nature Portfolio guidelines for submitting code & software for further information.

Data

Policy information about availability of data

All manuscripts must include a data availability statement. This statement should provide the following information, where applicable:

- Accession codes, unique identifiers, or web links for publicly available datasets
- A description of any restrictions on data availability
- For clinical datasets or third party data, please ensure that the statement adheres to our policy

Raw Human data has been deposited in the European Genome-Phenome Archive (EGA) under EGA accession number EGAD50000000410 for the scATAC-seq, nanoCT-seq, sc multiome and hOPCs microC data, EGAD50000001542 for the scRNA-seq from the human prefrontal cortex biopsy and EGAD50000001535 for Bcells microC data. Browsable tracks are available at UCSC Genome Browser (https://cns-nanocuttag-atac.cells.ucsc.edu).

Research involving human participants, their data, or biological material

Policy information about studies with human participants or human data. See also policy information about sex, gender (identity/presentation), and sexual orientation and race, ethnicity and racism.

Reporting on sex and gender	The study used post-mortem human tissue from 3 regions of the CNS from 20 donors and was collected by the MRC Sudden Death Brain Bank in Edinburgh, Scotland. Consent was obtained from the donor's family at the time of collection. All ethical permits to use and handle the tissue are in place. The Sex of the donors is known and is included in the metadata (Supplementary Table 1).<br><br>The prefrontal cortex sample was selected from a cohort of patients with hydrocephalus, a 76 year old female, planning to undergo CSF diversion surgery either with a ventriculoperitoneal shunt placement (VP) or ventriculocisternostomy (VCS) and without diagnosed CNS malignancy, hematoma, infection, or inflammation. The study was approved by the Stockholm Region's ethical committee (2016/1062-31/2 and 2018/843-32).
Reporting on race, ethnicity, or other socially relevant groupings	n/a - this information was not collected
Population characteristics	Age of the donors at the time of death is included in the metadata (Supplementary Table 1)
Recruitment	Archival tissue is from non-diseased post-mortem individuals. In the study including the sample from the 76 year old female, patients and relatives received oral and written information about the study before inclusion and provided signed informed consent on hospital admission. Patients not wishing to participate or who were not capable of understanding information or to provide signed informed consent were excluded. Patient data and samples were anonymized for the research group.
Ethics oversight	Post-mortem tissue was obtained from the MRC Sudden Death Brain Bank in Edinburgh with full ethical approval (16/ES/0084) and consent. Work in Sweden was performed under the ethical permit 2016/589-31, with amendment 2019-01503, granted by the Swedish Ethical Review Authority (EPN).<br><br>The prefrontal cortex sample was selected from a cohort of patients with hydrocephalus, a 76 year old female, planning to undergo CSF diversion surgery either with a ventriculoperitoneal shunt placement (VP) or ventriculocisternostomy (VCS) and without diagnosed CNS malignancy, hematoma, infection, or inflammation. The study was approved by the Stockholm Region's ethical committee (2016/1062-31/2 and 2018/843-32).

Note that full information on the approval of the study protocol must also be provided in the manuscript.

Field-specific reporting

Please select the one below that is the best fit for your research. If you are not sure, read the appropriate sections before making your selection.

☒ Life sciences ☐ Behavioural & social sciences ☐ Ecological, evolutionary & environmental sciences

For a reference copy of the document with all sections, see nature.com/documents/nr-reporting-summary-flat.pdf

Life sciences study design

All studies must disclose on these points even when the disclosure is negative.

Sample size	No sample size calculation was performed to pre-determine sample sizes.<br><br>The sample sizes for snATAC-Seq were in the same range of a previously published snRNA-Seq study (Seeker et.al. 2023).<br>Nano-CUT&Tag is performed for the first time in human CNS archival tissue. Three cervical spinal cord and three cortical frozen archival tissue samples from a total of four donors were used.<br>Micro-C was performed on 3 biological replicates in hOPCs and memory B-cells.

Data exclusions	12 of the original 60 tissue samples were not processed due to low RIN values assessed previously (Seeker et.al. 2023).
Replication	Comparisons with available single cell epigenomics and transcriptomic datasets were performed in the case that ortogonal datasets were available.
Randomization	Samples were pseudo-randomized for processing during experiments and loading on the 10x microfluidics chip. Care was taken to include samples from at least 2 donors, and spanning all three regions per experiment. Sequencing libraries were randomized for sequencing.
Blinding	Data collection and analysis were not performed blind to the conditions of the experiments

Reporting for specific materials, systems and methods

We require information from authors about some types of materials, experimental systems and methods used in many studies. Here, indicate whether each material, system or method listed is relevant to your study. If you are not sure if a list item applies to your research, read the appropriate section before selecting a response.

Materials & experimental systems

n/a	Involved in the study	
☐	☒	Antibodies
☐	☒	Eukaryotic cell lines
☒	☐	Palaeontology and archaeology
☒	☐	Animals and other organisms
☒	☐	Clinical data
☒	☐	Dual use research of concern
☒	☐	Plants

Methods

n/a	Involved in the study	
☒	☐	ChIP-seq
☐	☒	Flow cytometry
☒	☐	MRI-based neuroimaging

Antibodies

Antibodies used	The following antibodies were used in the multimodal nano-CT experiments: mouse anti-H3K27me3 (Abcam, Ab6002), rabbit anti-H3K27ac (Abcam Ab177178).
Validation	All antibodies used in this study have been validated and tested by the provider company and/or have been cited by other authors, references are available on the web page of the provider company.

Eukaryotic cell lines

Policy information about cell lines and Sex and Gender in Research

Cell line source(s)	human iPS-derived OPCs (provided by Steven Goldman, not commercially available), with the protocol described in Wang. et.al.2013, from the C27 IPS line (https://www.nature.com/articles/nbt.1529)
Authentication	Human iPS-derived OPCs were authenticated by Steven Goldman's lab according to their standard procedure, and single cell RNA-Seq data in the Goldman lab and in the Castelo-Branco lab confirmed their
Mycoplasma contamination	Routine mycoplasma testing was performed by the Goldman lab. Cells were tested monthly for mycoplasma (testing by PCR) and tested prior to freezing and post-thawing.
Commonly misidentified lines (See ICLAC register)	n/a

Plants

Seed stocks	n/a
Novel plant genotypes	n/a
Authentication	n/a

Flow Cytometry

Plots

Confirm that:

☒ The axis labels state the marker and fluorochrome used (e.g. CD4-FITC).

☒ The axis scales are clearly visible. Include numbers along axes only for bottom left plot of group (a 'group' is an analysis of identical markers).

☒ All plots are contour plots with outliers or pseudocolor plots.

☒ A numerical value for number of cells or percentage (with statistics) is provided.

Methodology

Sample preparation	Trypsination and resuspension in PBS
Instrument	SONY MA900
Software	FlowJo_v10.8.1
Cell population abundance	85-90% of total cells
Gating strategy	identification of viable cells using SSC-A and FSC-A. 2: identification of single cells using FSC-A and FSC-H. 3: identification of BFP and mCherry transduced cells

☒ Tick this box to confirm that a figure exemplifying the gating strategy is provided in the Supplementary Information.
